# Evidence for the clinical effectiveness of decongestive lymphoedema treatment for breast cancer–related arm lymphoedema, a systematic review

**DOI:** 10.1007/s00520-024-08759-x

**Published:** 2024-08-02

**Authors:** Eunice Jeffs, Emma Ream, Cath Taylor, Arnie Purushotham, Debra Bick

**Affiliations:** 1 King’s College London and Guy’s & St Thomas’ NHS Foundation Trust, London, UK; 2 School of Health Sciences, University of Surrey, Guildford, UK; 3 School of Cancer and Pharmaceutical Sciences, King’s College London, London, UK; 4 Warwick Clinical Trials Unit, Warwick Medical School, University of Warwick, Coventry, UK

**Keywords:** Systematic review, Breast cancer, Lymphoedema/lymphedema, Decongestive lymphoedema treatment, Outcomes

## Abstract

**Purpose:**

Early treatment is advised for breast cancer–related arm lymphoedema (BCRL), a common sequelae of breast cancer treatment. Expert guidance recommends two-phase decongestive lymphoedema treatment (DLT), although evidence is lacking for current treatment protocols and UK women are routinely offered self-treatment with hosiery.

This systematic review considered evidence regarding treatment of early BCRL, that is, within 12 months of developing BCRL.

**Methods:**

A systematic review of evidence for clinical effectiveness of DLT for women with less than 12-month BCRL duration (early BCRL) was undertaken using the Joanna Briggs Institute (JBI) method. Studies included women with < 12-month or mean < 9-month BCRL duration; some studies reported only one eligible group. The original search was conducted in 2016 and updated in 2018 and 2022. Methodological quality of identified studies was assessed using JBI critical appraisal instruments. Outcomes of interest were extracted with eligible results displayed in narrative and tabular format. Strength of evidence was rated using the GRADE system.

**Results:**

Seven trials and three descriptive studies provided weak evidence (grade B) for effectiveness of DLT for early BCRL. Heterogeneous protocols limited comparison of findings. There was no evidence for the most effective treatment or treatment combination or optimal frequency or duration of treatment.

**Conclusion:**

There is no evidence to justify change in current lymphoedema treatment, whether self-treatment with hosiery (UK) or two-phase DLT (other countries). Further research for the early BCRL population is required.

**Implications for cancer survivors:**

Women with early BCRL require early and effective treatment although this updated review shows there is still no evidence for what that treatment should be.

**Supplementary Information:**

The online version contains supplementary material available at 10.1007/s00520-024-08759-x.

## Background

Breast cancer–related arm lymphoedema (BCRL) is a common sequelae of breast cancer treatment with a significant physical and psychosocial impact on women and their families [1, 2]. BCRL typically presents as arm swelling, whole or part, often extending into hand and fingers and sometimes the adjacent trunk quadrant, including breast lymphoedema. Symptoms may include heaviness, with reduced manual dexterity affecting activities of daily living sometime require work environment modifications or change of employment [3–5]. Women with BCRL typically experience greater psychological morbidity and psychosocial impact than those with breast cancer alone [2, 6]. For many women, swelling may initially subside with rest and elevation. For others, symptoms become more severe and increasingly difficult to treat, with firmer and fibrosed subcutaneous tissues and increased risk of infection due to reduced lymphatic drainage [7–10].

In recent years, prophylactic lympho-vascular anastomotic surgery and less aggressive axillary surgery (sentinel lymph node biopsy) and radiotherapy protocols have minimised axillary treatment and reduced the incidence and presenting severity of BCRL [11]. However, a pooled BCRL incidence of 14.29% (95%CI 13.79–14.79) suggests that of the 55,000 new cases of invasive cancer diagnosed each year in the UK at least 7000 will develop BCRL within 2 years following cancer treatment, with others developing BCRL many years later [12–14].

Lymphoedema remains a physical and psychosocial problem regardless of the amount of visible swelling or whether symptoms are mild, moderate or severe [15]. Although women are routinely advised to access early treatment should swelling occur, evidence for effectiveness of initial BCRL treatment is lacking. International consensus has for the past 30 years recommended two-phase decongestive lymphoedema treatment (DLT), a combination of conservative treatments also known as complex decongestive treatment (CDT), to reduce and control lymphoedema and avoid the complication of cellulitis [16–18]. While several studies have shown that women who receive initial intensive-DLT followed by maintenance self-treatment with hosiery will achieve better outcomes than those self-treating with hosiery alone, particularly for those with mild and recent BCRL onset [19–22], Dayes et al. [23] found no significant difference in outcomes (*n* = 105). Systematic reviews have concluded there is weak evidence for the effectiveness of DLT although insufficient to inform an optimal DLT package due to the wide range of treatment protocols, programme duration and variation in study population severity [24, 25]. Most reviews noted a predominance of underpowered studies, heterogeneity of treatment protocols and measured outcomes and lack of follow-up monitoring long-term effects [24–26].

International awareness of BCRL has greatly improved since an early systematic review concluded the major limiting factors for BCRL treatment were lack of awareness of the problem and insufficient resource to manage it [27]. BCRL is no longer seen as a neglected or rare condition, with women now routinely informed about self-management strategies to minimise the risk of BCRL and signposting to ensure they no longer wait months or years to access treatment [28]. However, despite the existence of many UK lymphoedema services, lymphoedema treatment capacity remains a problem, and women are routinely offered self-treatment with hosiery although the impact of this approach on treatment outcomes has not been addressed [16, 18, 29]; UK practitioners struggle to provide appropriate care to increasing caseloads of individuals with all types of lymphoedema and complex comorbidities [30, 31]. In contrast, other countries consider two-phase DLT with initial intensive-DLT to be standard care despite reporting limited service capacity for providing intensive-DLT [32] or MLD [33].

Research into BCRL management has for many years focused on surveillance and testing whether prophylactic interventions for subclinical oedema will reduce the incidence of BCRL [34]. A previous review found that evidence for the effectiveness of initial treatment for women with early *established* BCRL, that is, less than 12-month duration, hereafter referred to as ‘early BCRL’, is lacking [35] yet this is not considered a research priority.

The objective of this updated systematic review was to (1) identify the effect of DLT on excess arm volume (EAV) and patient-reported outcomes for women with early BCRL and (2) determine whether four years after the original review [35], there was still the need for a definitive trial; it is good practice to periodically update systematic reviews and report changes to findings [36]. The definition of early BCRL as < 12-month duration was chosen to exclude women with chronic changes to their affected arm, such as fibrosis and thickening, which would be more difficult to treat.

## Method

### Literature searches and inclusion criteria

**Fig. 1:**
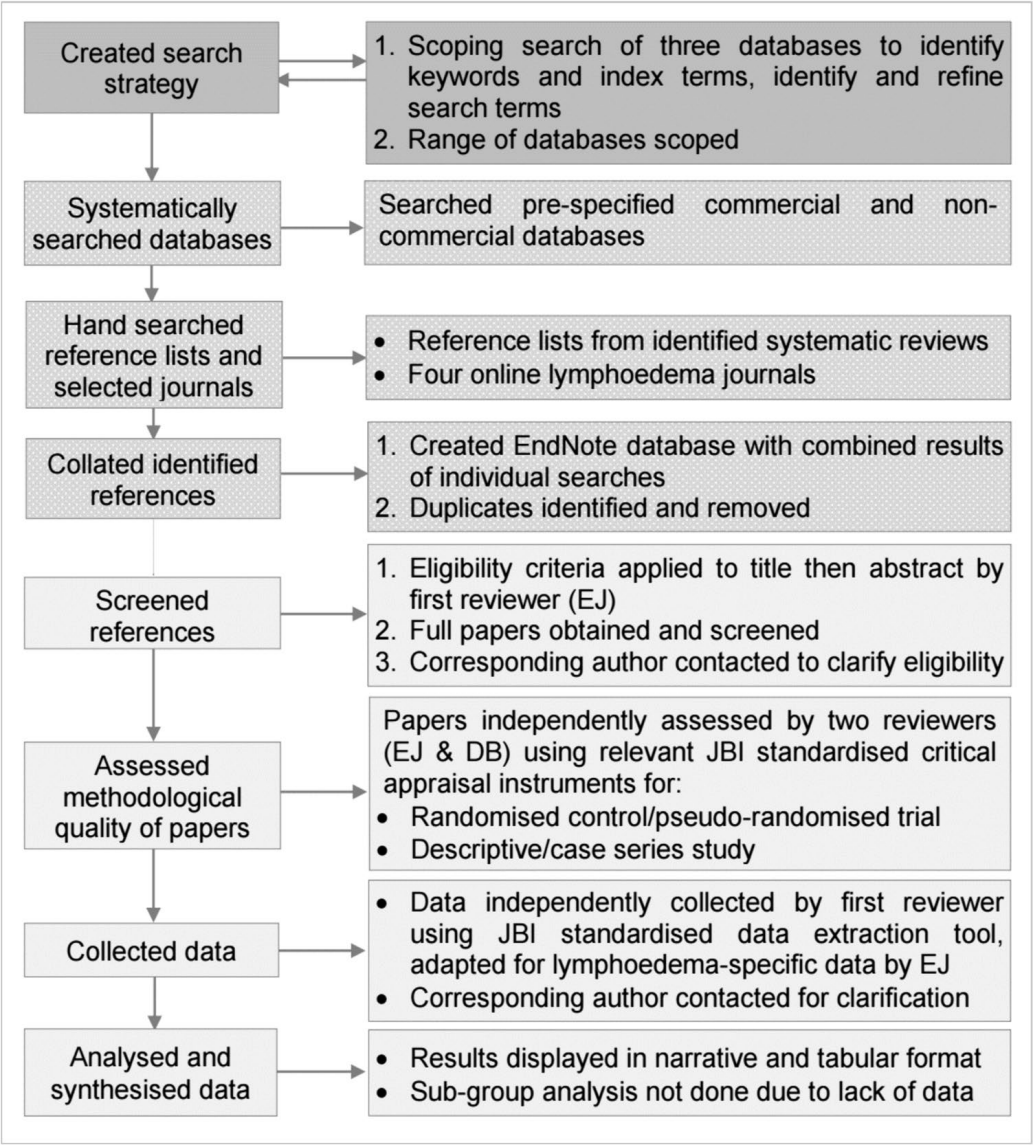
Overview of systematic review method

Figure 1 shows the review process which was conducted according to the published protocol [37, 38], PROSPERO (CRD42015015843), as reported in the PRISMA checklist (Online Resource 1). The JBI review method was chosen because it allowed inclusion of uncontrolled quantitative studies and it was expected that relevant lymphoedema research studies could be of non-experimental design [39–41].

Eligibility criteria and keywords (Table 1) were developed using the PICO framework and JBI terminology (type of Population, Intervention, Comparator, Outcome, Study) [40, 41]. The final search strategy combined variant terms for ‘lymphoedema’ and ‘breast cancer’ in title, abstract, subject heading and keyword (Online Resource 2). The strategy was deliberately broad as attempts to refine searches omitted potentially relevant papers. The list of included databases (see Online Resources 2 and 3) was also broad, including a wide range of medical and nursing databases, to identify relevant black and grey literature and non-English language publications with an English language abstract, with no date limits applied; early lymphoedema papers were published in journals which were not index linked.

**Table 1:** Eligibility criteria, structured using type of Population, Intervention, Comparison, Outcomes and Studies (PICOS)

	Inclusion	Exclusion
Population	Women with unilateral BCRL who received lymphoedema treatment < 12 months, or mean < 9 months following swelling onset	• Men • Other forms of BCRL • Bilateral or contralateral BCRL • Mean BCRL duration ≥ 9 months at initial lymphoedema treatment • BCRL of only hand, breast, trunk • Women ‘at risk’ of BCRL • Current breast cancer treatment (hormone therapy excepted) • Lower limb lymphoedema
Intervention	Any practitioner or patient-provided DLT to reduce arm lymphoedema, given < 12 months following BCRL onset: • Course of CDT or intensive-DLT • Compression as CB, hosiery or IPCT • MLD • Exercise, e.g. resistance training • LLLT	• Studies reporting interventions intended to prevent BCRL • Surgical interventions • Drug therapy • Treatment of progressive lymphoedema due to uncontrolled active cancer • Safety assessment of interventions • Evaluation of single therapy session (e.g. MLD, bandaging) • Comparative assessment technique
Comparator	• Another form of practitioner or patient-provided lymphoedema treatment, placebo, no treatment • Within-group comparison	
Outcome	Treatment effect: • Clinical outcome: relative change in EAV, change in tissue fluid measured by BIS or TDC • Patient-centred outcome: including HRQOL, heaviness sensation in affected arm, limb function, patient-perceived benefit, treatment satisfaction	Studies not reporting one of the following: relative change in EAV, tissue fluid, appropriate psychosocial or patient-reported outcome measure, patient value of treatment
Studies	• Experimental study designs: RCT, non-RCT, quasi-experimental studies • Descriptive (uncontrolled) studies, including before-and-after studies, whether prospective or retrospective	Qualitative studies

The original searches in July 2016 [35] were updated on 6 July 2018 following the original protocol although without searching grey literature [37, 38]. The most recent search, on 8 September 2022, was limited to PubMed, CINAHL and AMED databases which was a pragmatic decision to manage the time-consuming process of updating the review by searching those databases considered most likely to capture all relevant literature from journals addressing medicine, nursing and professions allied to medicine; the review protocol was otherwise unchanged (Online Resource 3). Four lymphoedema journals (EJLRP, J.Phlebo.Lymphol, Lymphology, J Lymphoedema) were hand-searched on each occasion (Online Resource 4), and reference lists from contemporary systematic reviews of BCRL treatment were checked for additional references, including Lasinski et al. [42] in 2016 and Davies et al. [43] and Marchica et al. [44] in 2022. All search results were electronically imported into EndNote with duplicate references removed using the automated duplicates finder and by manual sorting. The remaining records were combined into a single EndNote reference library (version X9).

One reviewer (EJ), an expert lymphoedema practitioner and researcher, screened records against the review eligibility criteria (Fig. 1): firstly, to remove titles describing other populations and interventions not of interest; secondly, to remove papers without an abstract and abstracts which did not include the population, interventions and outcomes of interest; finally, to remove full papers not fulfilling the eligibility criteria (Table 1), and examining at this stage those papers with abstracts lacking sufficient detail for exclusion. Eligibility of non-English papers was confirmed using online translation tools and bilingual colleagues; no formal translation was required. Due to a lack of studies with the population of interest, studies were included which separately reported a subgroup of the whole population or one study group with < 12-month or mean < 9-month BCRL duration which would keep to a minimum the inclusion of women with duration ≥ 12-month (chronic BCRL). The number of references excluded at each stage was recorded with reasons documented only for those excluded at full paper screening. Corresponding authors were contacted where full papers contained insufficient detail to determine eligibility; papers were excluded if contact details were missing or corresponding authors did not respond.

### Methodological assessment and data extraction

Two reviewers (EJ, DB) independently assessed included papers using standardised JBI critical appraisal tools to determine how each study addressed the possibility of bias in its design, conduct and analysis [35, 41]. A lymphoedema definitions list (Online Resource 5) was developed (EJ) to guide assessment by the secondary reviewer (DB). Differences of opinion were resolved through discussion, with no input required from a third independent reviewer (ER). Methodological limitations were reported rather than excluding poor quality papers.

Relevant outcomes were extracted (EJ) using the JBI standardised data extraction tool (Online Resource 6) adapted (EJ) to capture sufficient data to describe lymphoedema setting, population, interventions and outcomes of interest [41]. Corresponding authors were contacted where data were missing or unclear. Due to wide heterogeneity of interventions and outcomes of interest, findings could not be synthesised but are instead displayed in narrative and tabular format according to reported study intervention with summary statistics; relevant missing data are reported. Certainty of evidence was rated using the GRADE system with a strong recommendation (grade A) for health benefits indicating robust evidence of good quality and a weak recommendation (grade B) indicating some benefit with poor quality evidence [45]. There were insufficient data to perform two planned subgroup analyses: (1) previous lymphoedema treatment and (2) severity of swelling at baseline.

## Results

### Study selection

**Fig. 2:**
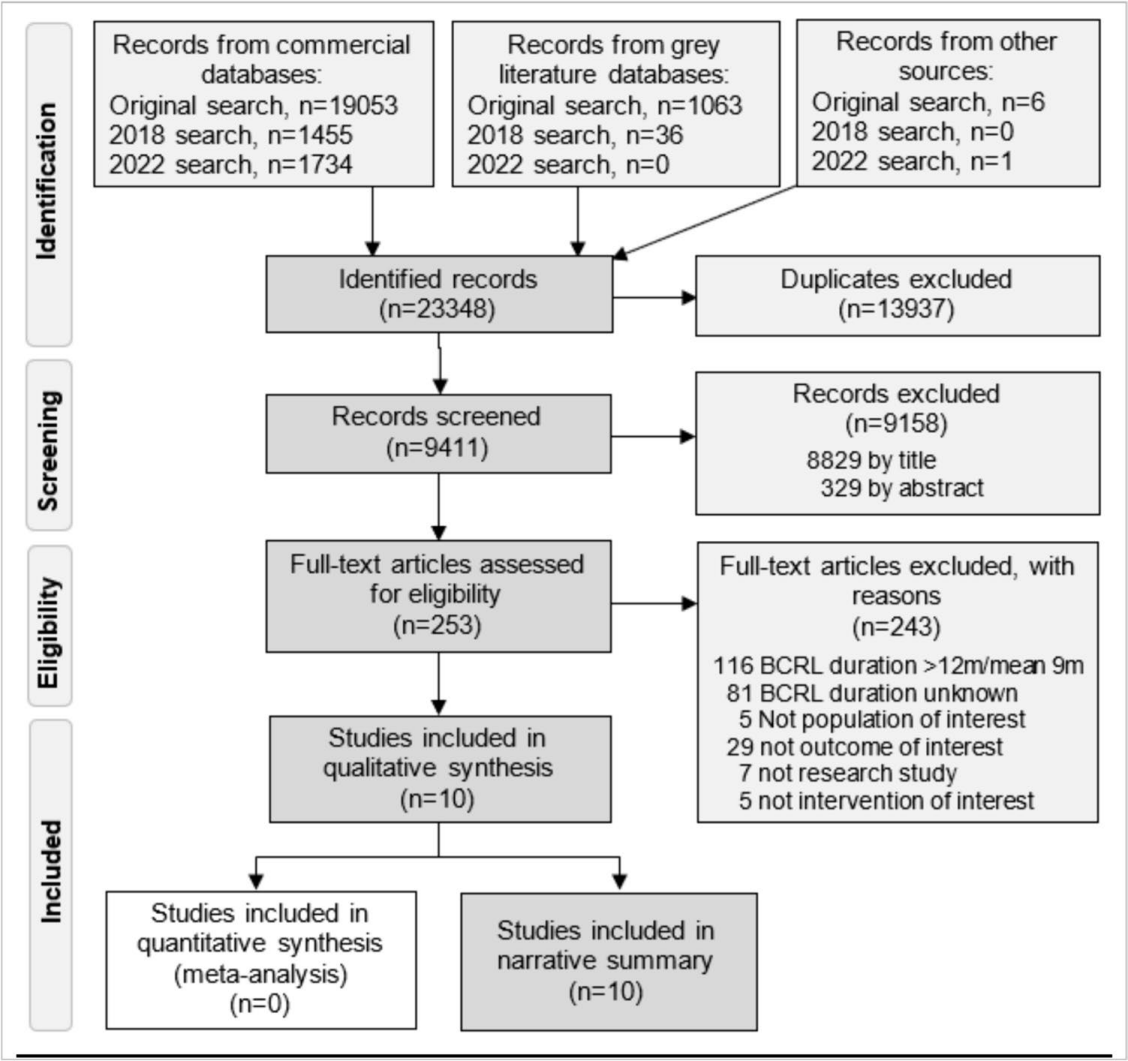
PRISMA flow diagram of search and study selection process

A total of 23,348 references were identified from three searches (Fig. 2), with most excluded as duplicates (*n* = 13,937, 60%) and by screening titles (*n* = 8829, 38%). Excluded abstracts (*n* = 329) included conference abstracts (*n* = 33) without a full publication. Excluded full papers (*n* = 243) are reported with reasons in Jeffs et al. [35] for the original search (*n* = 160) and in Online Resource 7 for updated searches (*n* = 83). Many studies did not report BCRL duration.

Only 10 studies fulfilled the eligibility criteria, six from the original search, one from 2018 and three from 2022. Another paper included in the original review [35] was excluded here because their findings were presented as figures and, due to lack of clear labelling, could not be compared with other studies [46]. The included studies were six RCT [21, 23, 47–50], one quasi-controlled study [51], one prospective uncontrolled study [52] and two retrospective case review studies [53, 54].

### Methodological assessment

All studies had significant areas of bias, with controlled studies scoring 6–10 out of 13 items (Table 2) and uncontrolled studies 3–7 out of nine items (Table 3). Only two trials described both randomisation method and allocation concealment [21, 23]. Although five trials blinded their assessors [21, 47–49], participant and therapist were not blinded to allocation despite three trials testing interventions with potential for blinding participants: bandage method, exercise programme [49–51]; there is no sham version of manual lymph drainage (MLD), self-lymphatic drainage (SLD) or two-phase DLT [21, 23, 47, 48]. Sample sizes for this review were small (*n* = 24–57), affecting precision of estimates and increasing the possibility of a type II error. Six trials reported attrition (5–40%), for mostly study-related reasons [21, 23, 48, 50, 51], although only one trial conducted sensitivity analyses [23].

**Table 2:** Results of critical appraisal based on Joanna Briggs Institute (JBI) tool for appraising randomised controlled trials (RCT)

*Citation*	*Abbasi, 2018 *[51]	*Bahtiyarca, 2019 *[47]	*Dayes, 2013 *[23]	*Gradalski, 2015 *[48]	*Kim, 2010 *[49]	*McNeely, 2004 *[21]	*Oh, 2019 *[50]	*Total (n* = *7)*
1. True random allocation	N	Y	Y	Y	?	Y	Y	5
2. Allocation concealed	N	?	Y	?	?	Y	?	2
3. Groups similar at baseline	Y	Y	Y	Y	Y	Y	Y	7
4. Participants blinded	N	N^1^	N^1^	N^1^	N	N^1^	N	0
5. Therapist blinded	N	N^1^	N^1^	N^1^	N	N^1^	N	0
6. Assessor blinded	?	Y	Y	Y	Y	Y	?	5
7. Treated identically	Y	Y	Y	Y	Y	Y	Y	7
8. Follow-up complete	Y	N	Y	Y	Y	Y	?	5
9. ITT per-protocol analysis	N	N	N	N	Y	N	N	1
10. Same outcomes measured	Y	Y	Y	Y	Y	Y	?	6
11. Reliably measured	Y	Y	Y	Y	Y	Y	Y	7
12. Statistical analysis	Y	Y	Y	Y	Y	Y	Y	7
13. Trial design	?	Y	Y	Y	?	Y	?	4
Total ‘Y’	6/13	8/13	10/13	9/13	8/13	10/13	5/13	

Key: *Y* yes, *N* no, *?* unclear, *ITT* intention to treat

^1^No sham treatment possible

**Table 3:** Results of critical appraisal based on JBI tool for appraising quasi-experimental, non-randomised studies

*Citation*	*Haghighat, 2013 *[52]	*Hwang, 2013 *[54]	*Michopoulos, 2021 *[53]	*Total (n* = *3)*
1. Clarity regarding cause and effect	Y	Y	Y	3
2. Groups similar at baseline	N/A	Y	N	1
3. Groups treated identically	N/A	Y	Y	2
4. Control group	N	N	N	0
5. Multiple pre- and post-measurements	N	Y	Y	2
6. Follow-up complete	Y	?	Y	2
7. Same outcomes measured	N/A	Y	Y	2
8. Reliably measured	?	Y	Y	2
9. Appropriate statistical analysis	Y	Y	Y	3
Total ‘Y’	3/6	7/9	7/9	

Key: *Y* yes, *N* no, *?* unclear, *N/A* not applicable

All trials reported comparable study groups at enrolment and, except for their intervention of interest, treated women identically and according to their study allocation. Outcomes included in this review were measured using reliable methods, although findings were excluded for two studies which reported absolute volume [50] or only the affected arm [49]. Only one study [21] reported training assessors which would reduce measurement bias although two studies used experienced lymphoedema practitioners [48, 50]. However, heterogeneity of outcomes and measurement methods made comparisons difficult, and studies excluded from their analysis those women who did not complete allocated treatment or subsequent study follow-up.

The two retrospective case review studies had low risk of bias [53, 54]. The prospective uncontrolled study [52] lacked sufficient detail to ensure reproducibility of reported outcome measurements, used a non-validated tool to measure self-reported symptoms and did not follow study outcomes beyond the immediate post-intervention period.

### Characteristics of included studies

Table 4 shows an overview of key characteristics for each study with findings reported for only the eligible group, excluding findings for intervention and comparator groups with > 12-month or mean > 9-month BCRL duration.

**Table 4:** Characteristics of included studies, reporting study design, participants, interventions, outcomes and follow-up

Study, design, place, time	Outcomes, follow-up	Participants	Interventions, delivered by	Duration, frequency	Comments
Abbasi et al., 2018 [51] Quasi-RCT, non-randomised. Hospital rehabilitation clinic, Iran. 8 months, 2013	EAV, absolute volume, relative change in %EAV. Reported pre-post intervention and 6-week post-intervention. Assessor role unclear; data collected by trained researcher and trained therapist.	Recruited 38 women with BCRL, defined as ≥ 200 cc difference between arms. Analysed 31 with 16 included in this review. **CDT group** (*n* = 16), age 52.06 ± 10.18 years. Mean BCRL duration 8.56 ± 7.89 months. Baseline %EAV and severity not reported. BMI 29.03 ± 4.31 *(CDT* + *relaxation group (n* = *15) excluded as 10.76* ± *14.08 months BCRL duration)*	**CDT**: (study comparator) Phase-1: CB, MLD, rehabilitation exercise, skin and nail care; details not specified. Delivery personnel not reported. Phase-2: self-treatment with hosiery (type and strength not reported), SLD, unspecified exercise, skin and nail care; multi-layer bandaging at night. *(CDT* + *relaxation, study intervention, excluded)*	**CDT**: Phase-1: daily 60 min treatment for 3 weeks (18 sessions). Phase-2: daily self-treat post-intervention, hosiery wear time not specified.	Excluded women with prior BCRL treatment. No sample size calculation. Routine care maintained during study. Attrition (*n* = 3). Insufficient detail to replicate study without making assumptions. Also measured anxiety and depression (HADS) but not outcome of interest.
Bahtiyarca et al., 2018 [47] RCT. Hospital lymphoedema clinic, Turkey 2 years 1 month, 2015–2017	EAV, absolute volume, volume difference. QOL (SF-36) and functional status (QuickDASH), Turkish versions. Reported pre-post intervention and 6-months. Measured by researcher blind to allocation, training not reported.	Recruited 40 women with BCRL, defined as > 10%EAV. Analysed 24, all included in this review. **CB + SLD** (*n* = 10), age 55.2 ± 7.2 yr. *Md* 1 (1–36) month BCRL duration, baseline 25.1 ± 11.3%EAV, ISL severity stage 1 (*n* = 2), stage 2 (*n* = 8). BMI 30.88 ± 3.62 **CB** (*n* = 14), age 61.64 ± 11.69 yr. *Md* 2 (1–12) months BCRL duration. Baseline 24.7 ± 15.8%EAV, ISL stage 1 (*n* = 3), stage 2 (*n* = 11). BMI = 32.73 ± 5.80	**CB + SLD**: Phase-1: CB applied by clinician, SLD done before CB, skin care, physical exercise. Clinician training not reported. Phase-2: self-treatment with custom-fit garments, 20–30 mmHg (class 2), worn daytime only. **CB**: Phase-1: as above, without SLD Phase-2: as above.	**CB + SLD**. Phase-1: daily treatment (time n.d.) 5 × /wk for *Md* 5 (4–8) weeks duration; SLD 10–15 min daily; CB worn 23 h/day. Phase-2 self-treatment, hosiery worn daily daytime. **CB**. As above, without SLD. Phase-1 for *md* 6 (4–20) weeks.	Previous BCRL treatment not study exclusion criteria. No sample size calculation. Attrition, *n* = 16, 6 from CB and 10 from CB + SLD. Insufficient detail to replicate study. Also measured anxiety and depression (HADS) but not an outcome of interest.
Dayes et al., 2013 [23] RCT, 6 regional cancer centres, Canada. 6 years, 2003–2009. Data for relevant subgroup provided by corresponding author	EAV, %EAV and change in EAV and %EAV. QOL (SF-36) and functional status (DASH). Reported pre-post intervention, 6 weeks, 12 months; ‘n’ varied per visit. Measured by study assessor blind to study allocation; training not reported.	Recruited 103 women with BCRL, defined as > 10%EAV. Analysed 95, of whom 58 with < 1-year BCRL duration included in this review. **DLT group** (*n* = 32) *Md* age 61 yrs (36, 83), BMI 30, baseline %EAV mean 23 ± 12, severity 10 < 20% (*n* = 15), 20 < 30% (*n* = 11), ≥ 30% (*n* = 6). **Self-treatment group** (*n* = 26), *Md* 59 yrs (46, 76), BMI 30, baseline %EAV mean 21 ± 7, severity 10 < 20% (*n* = 15), 20 < 30% (*n* = 9), ≥ 30% (*n* = 2).	**DLT group**: Phase-1: CB (detail not reported), MLD (Vodder or Foldi method), unspecified exercise, skin care, maintain healthy body weight. Phase-2: Armsleeve (30–40 mmHg) worn waking hours, unspecified exercise, skin care, maintain healthy body weight. **Self-treatment group**: Self-care, as phase-2 above.	**DLT group**: Phase-1: daily 5 × /wk for 4 weeks (20 sessions), including CB worn 23 h/day, 1-h MLD, by trained therapist, taught self-CB for weekend use. Phase-2: Self-treat with hosiery worn 12 h/day. **Self-treatment group**: as Phase-2 treatment.	Sample size calcs, severity data and attrition (*n* = 8) not separately provided for subgroup with BCRL < 12 months. Excluded if previous intensive-DLT (ever) or armsleeve within past 1-month. Lacking details to replicate CB. Recruitment target achieved. 95% of target analysed.
Gradalski et al., 2015 [48] RCT, unspecified treatment facility in Poland. 2 year 8 months, 2010–2012.	EAV, relative EAV change. BCRL-specific QOL non-validated questionnaire. Reported pre-post intervention and 6 month outcomes. Therapist-assessor blind to measurements, not study allocation; no training reported.	Recruited 60 women with BCRL defined as ≥ 20%EAV. Analysed 51 with 25 included in review. **DLT group** (*n* = 25) with mean 8.3 ± 7.2 months BCRL duration, age 61.2 ± 9.2 years, BMI 30 ± 6.3; baseline %EAV and severity not reported. *(CB group (n* = *26) excluded with mean BCRL duration 9.4* ± *10.2 month)*	**DLT group**: Phase-1: CB (details provided) plus Vodder II method MLD, specified exercises and deep breathing while wearing CB. Phase-2: custom-made flat-knit armsleeve (strength not specified), exercise, arm and skin care. *(CB, comparator, excluded)*	**DLT group**: Phase-1: 2 weeks 5 × /week daily CB and 30 min MLD by experienced physiotherapists, plus 15 min exercise, 10 sessions. Phase-2: daily hosiery and twice-daily exercises as phase-1.	Women excluded if prior BCRL treatment. Sample size calcs, achieved target recruitment. Attrition *n* = 5 (plus 4 from excluded group). Provided sufficient data to replicate study. Visual analogue scale (VAS) for satisfaction with treatment, not reported.
Haghighat et al., 2013 [52] Descriptive study, prospective case series. Breast Cancer Centre BCRL Clinic, Iran 1 year, 2008. Corresponding author provided data.	EAV, relative EAV change, reported pre-post intervention only. Measurement method and assessor not reported. Heaviness (VAS) not reported for subgroup in this review. Other outcomes not of interest.	Recruited 137 women with BCRL; definition not provided. Analysed 137 with 60 with BCRL duration < 12 months included in this review. No characteristics provided for the subgroup. Total population (*n* = 137) was aged 53.5 ± 10 years; BMI 29.7 ± 4.7; baseline %EAV and severity not reported.	**DLT group**. Phase-1: CB using ‘bandage set’, Vodder method MLD, unspecified remedial exercises, skin care. No phase-2 CDT. Therapist not reported. **Comparator**: none.	**DLT group**. 2–3 weeks 5 × /week, daily treatment with CB and 45 min MLD (10–15 sessions). Criteria not reported for deciding number of treatment sessions.	Excluded women with prior BCRL treatment. No justification for sample size. No attrition reported. Findings for women with ≤ 12-month BCRL duration provided as personal communication. Lacks sufficient detail to replicate bandaging.
Hwang et al., 2013 [54] Descriptive study, review retrospective case series. Department of Physical and Rehabilitation Medicine, South Korea. 10 years 10 months, 2001–2011.	EAV (Perometer measurement); %EAV, relative EAV change. Reported pre-post intervention and at 24 months. Assessor experienced in lymphedema treatment; training not reported.	Of 57 women retrospectively reviewed, a subgroup with < 20%EAV (*n* = 32) had mean 8.5 ± 6.6 months BCRL duration, age 47.5 ± 9.8 years, BMI 25.9 ± 4.8, baseline %EAV 11.4 ± 5.0, ISL severity not reported. BCRL definition not provided. *(Subgroup with* ≥ *20%EAV had mean 27.5* ± *31.5 BCRL duration so excluded)*	**DLT group**. Phase-1: CB + MLD (methods not specified), unspecified remedial exercises, skin care. Phase-2: daily self-care with armsleeve (strength not reported), SLD (taught by practitioner), skin care, unspecified remedial exercises, plus 3 × /week night-time self-CB (taught by practitioner). Practitioners: skilled BCRL-certified physiotherapists. **Comparator**: none.	**DLT group**. Phase-1: 2 weeks daily treatment, 5 × /week (Mon–Fri), with 1 h MLD (10 sessions). Phase-2: daily daytime hosiery and other self-treatment plus at least three times weekly nighttime self-bandage.	Number of women with previous BCRL treatment not reported. No justification provided for the sample size. Data collected over long period. Insufficient detail to replicate intervention (CB, MLD, exercise).
Kim et al., 2010 [49] RCT. Department of Rehabilitation Medicine, South Korea. 1 year, 2009	QOL (SF-36). Reported pre-post intervention only. Interviewer blinded to treatment allocation; training of assessor not reported. Arm volume (circumference) excluded as only reported affected arm.	Recruited and analysed 40 women with BCRL, defined as > 2 cm difference between arms. **DLT + ARE** (*n* = 20), mean age 50.5 ± 10.55 years, BCRL duration 4.35 ± 12.91 months, BMI 25.1 ± 3.0 **DLT (non-ARE)** (*n* = 20), mean 50.9 ± 9.15 years, BCRL duration 5.24 ± 12.61 months, BMI 24.9 ± 2.7 Baseline %EAV and severity not reported.	**DLT + ARE** group. Phase-1: CB, MLD (method unspecified), specified remedial and breathing exercises. ARE program described. Phase-2: self-CDPT + ARE, with self-CB or hosiery, remedial exercises + ARE. **DLT, non-ARE** group. Phase-1-and-2, CDPT as above without ARE. Both groups treated by physical therapist.	**DLT + ARE** group. Daily CDPT plus 15 min ARE, 10 sessions over 2 weeks. Post-intervention self CDPT with CB or hosiery, remedial exercise. **DLT, non-ARE**. As intervention without ARE.	Skin care not reported. Sufficient detail provided to replicate exercise but lacks sufficient detail for CB and MLD. No sample size calculations. No attrition. No data provided regarding BCRL severity or number of women who received previous BCRL treatment.
McNeely et al., 2004 [21] RCT. Cancer rehabilitation unit, Canada. 1 year 1 month, November, 2000–2001.	EAV, %EAV, %change in EAV pre-post intervention only. Findings stratified as early BCRL (≤ 11 months) and chronic BCRL (> 11 months). Assessments by two independent trained physiotherapists blind to study allocation.	Recruited 50 women with BCRL defined as > 150 ml EAV. Analysed 45, mean age 58 years in MLD + CB group (*n* = 24), 63 years in MLD group (*n* = 21). BMI and baseline %EAV not reported, although severity reported by %EAV. Included 18 with BCRL duration ≤ 11 months in review: MLD + CB, *n* = 8; CB, *n* = 10. Characteristics not reported for this subgroup.	**MLD + CB group**. Vodder method MLD plus CB (method described), skin care, standard education re arm care (detail not provided). **CB group**. As above without MLD. Bandages applied by physical therapy assistant; MLD provided by Vodder-trained physiotherapist.	**MLD + CB group**. Daily CB plus 45 min MLD, 20 sessions over 4 weeks. **CB group**. As above, without MLD, 20 sessions over 4 weeks.	Included those wearing sleeve, but excluded if intensive-DLT in past 6-months. Sample size calcs, targets achieved. Attrition from whole group (*n* = 5). Sufficient detail to replicate CB and MLD. No subgroup data provided BCRL severity. Intra-rater and inter-rater reliability tested.
Michopoulos et al., 2021 [53] Descriptive study, retrospective cohort. Hospital Vascular Unit, Greece. 2 years 1 month, 2017–2019	EAV (circumference measurement), %EAV, %reduction EAV, reported pre-post intervention outcomes only. Assessor not reported.	Recruited and analysed 33 women with previously untreated BCRL; BCRL not defined. Included 12 women with BCRL duration ≤ 1 year, mean age 47.5 ± 8.0 years, baseline 28.8%EAV (24.7–43.9); ISL stage 1 (*n* = 3) and stage 2 (*n* = 9). Other 21 women excluded with BCRL > 1 year.	**DLT group**. Phase-1: Multi-layer short-stretch CB, skin care, lymphoedema rehabilitation exercise (not specified), MLD (Vodder method). MLD and CB administered by specialist physiotherapist. **Comparator**: none.	**DLT group**. Daily CB and 1-h MLD, 5 days/wk for 4 weeks (20 sessions).	No justification provided for sample size. Attrition from whole group, *n* = 5. Insufficient detail to replicate bandaging and exercise components. Described severity by %EAV for whole group. BMI not reported.
Oh et al., 2019 [50] RCT. Department of Rehabilitation Medicine, South Korea. Dates not provided.	Pre-post intervention only functional status (DASH). Treatment satisfaction 10 item, non-validated questionnaire. Assessor not reported. Other outcomes not included in review: absolute EAV (as not relative EAV), HRQOL (instrument and findings not reported).	Recruited 46 women with BCRL defined as > 2 cm or > 200 ml greater than unaffected arm. Analysed 42 women; **CB-spiral**: *n* = 21, age 57.3 ± 56.4 yrs, BMI 28.0 ± 8.3, BCRL duration 6.8 ± 7.5 months. **CB-spica**: *n* = 21, age 56.4 ± 7.9 yrs, BMI 26.1 ± 2.6, BCRL duration 8.4 ± 12.4 months. Baseline %EAV and severity not reported.	**CB-spiral**. CB applied with spiral technique; no other details reported. **CB-spica**. CB applied in criss-cross fashion; no other details reported. Otherwise, treatment the same for both groups; treatment details not reported although introduction suggests CDT. Treatment personnel not reported.	**CB-spiral**. 2 weeks duration, frequency not reported. **CB-spica**. 2 weeks duration, frequency not reported.	Sample size calcs. Attrition, *n* = 4; 2 women from each group. Insufficient detail to replicate the study. Lacks detail about conduct of study and severity of participants.

#### Participants

Results were available for 327 women with mean/median age from 47.5 to 61.6 years, although no study specifically addressed the population of interest. Four studies (*n* = 148) reported a subgroup with BCRL duration ≤ 12 months [21, 23, 52, 53], four studies (*n* = 138) had mean ≤ 9-month duration [47, 49, 50, 54] and two trials (*n* = 41) reported only one study group with mean ≤ 9-month BCRL duration [48, 50]. The number of women with prior treatment experience could not be determined as only four studies explicitly excluded women due to prior BCRL treatment [48, 51, 52]. Three studies did not address prior BCRL treatment and three excluded an unknown number of women who had received treatment within the previous 1–6 months [21, 23, 50].

The controlled studies defined BCRL by arm size difference using varying criteria, with four studies contributing a pooled mean baseline of 21%EAV [23, 48, 53, 54]. No descriptive study provided a BCRL definition. Three populations reported moderate BCRL, ≥ 20%EAV [23, 47, 53], and one reported mild BCRL, < 20%EAV [52]. Only two studies reported ISL lymphoedema severity stage [47, 53].

#### Study setting

Studies were conducted between 2000 and 2019 in countries with well-developed health care systems, mostly in Lymphoedema Units or Rehabilitation Departments associated with cancer services, with time periods varying from 8 months to 11 years. Studies were conducted in: Canada [21, 23], Iran [51, 52], South Korea [49, 50, 54], Turkey [47], Greece [53], Poland [48].

#### Interventions and comparators

The studies reported heterogeneous treatment protocols, intervention duration and components (Tables 4 and 5). Only two studies provided sufficient detail to replicate key components of both intervention and comparator groups [21, 48]. One trial [23] and three descriptive studies [52–54] provided intensive-DLT as the intervention, including compression bandaging (CB), MLD, exercise and skin care. A further four trials provided intensive-DLT to intervention and/or comparator group [21, 48, 49, 51]. There was heterogeneity of CB materials, application method, treatment duration (2–4 weeks) and post-intervention follow-up (0–104 weeks). Only two trials provided sufficient detail to replicate intervention and comparator [21, 48]. Detailed information regarding exercise and skin care was lacking from most studies.

**Table 5:** Summary of treatment components in each study

Study	Intervention period (w)	Follow-up period (w)	Compression bandaging	Compression sleeve	MLD	SLD	LLLT	Skin care	Remedial exercise	Unspecified exercise	Healthy weight	Other
Abbasi^1^ [51]	3	6	X n	o	X	o		X o	X o			Breathing
Bahtiyarca [47]	6 5	25 26	X X	o o		X		X X				
Dayes [23]	3 3	52 52	X	o X	X			X X		X X	X X	
Gradalski^2^ [48]	2	26	X	o	X			X o	X	o		
Haghighat^3^ [52]	2–3	0	X		X			X	X			
Hwang^3^ [54]	2	104	X n	o	X	o		X o	X o			
Kim [49]	2 2	6 6	X o X o	o o	X X				X o X o			Breathing; ARE Breathing
McNeely [21]	4 4	0 0	X X		X			X X				Arm care^4^ Arm care^4^
Michoupolos^3^ [53]	4	0	X		X			X	X			
Oh [50]	2 2	0 0	X X									

X = intervention, o = maintenance, n = nighttime use during self-treatment phase

^1^Study intervention (relaxation exercises) excluded

^2^Study comparator group (CB) excluded

^3^No control group

^4^Content of ‘arm care’ not recorded

All 10 studies provided CB, for 2–5 weeks, although only one trial [50] specifically addressed the use of CB without reporting sufficient detail regarding materials and other treatment components to replicate the intervention. Only two trials described the process sufficiently to replicate CB [21, 48]. Otherwise, CB was typically described as short-stretch without indicating the number or type of bandage and sub-bandage layers.

Two trials tested the use of MLD to determine whether treatment outcomes improved with intensive-DLT compared with CB alone [21, 48]. Protocols differed in number of sessions (10 and 20) and duration (30 and 45 min). Another six studies provided MLD within their treatment protocol [23, 49, 51–54], reporting only the session duration and treatment method, whether Vodder or Foldi, which should be sufficient for trained MLD practitioners to replicate the intervention.

One study tested whether SLD enhanced CB outcomes, providing 10–15 min SLD prior to bandaging, with SLD discontinued during the median 6-week post-intervention self-treatment period [47]. Two other studies included SLD only during the post-intervention self-treatment phase so were excluded from this analysis [51, 54].

One trial tested the benefit of adding 15-min active resistance exercise (ARE) to intensive-DLT compared with intensive-DLT alone, with ARE continued throughout the 6-week self-treatment phase [49]. Six other studies reported exercise done by both study groups [23, 47, 48, 52–54] although only one provided sufficient detail to replicate the exercises [48]. Another trial reported relaxation exercise as the intervention with findings excluded as the intervention group was not the population of interest [51].

### Outcomes

Outcomes of interest to this review included arm size, reported as relative change in EAV (Table 6) or pre- and post-intervention %EAV (Table 7); health-related quality of life (HRQOL) using the 36-item Short Form Health Survey (SF-36; RAND, USA) (Table 7); function using the Disabilities of the Arm, Shoulder and Hand (DASH) questionnaire and QuickDASH (Table 7); arm heaviness and patient-perceived benefit. Wide heterogeneity in study populations, interventions and measurement outcomes made meaningful comparison of data difficult and meta-analyses could not be performed.

**Table 6:** Results for studies reporting post-intervention relative (%) reduction in excess arm volume (EAV)^1^

Study	Intervention group	Duration (wks)	Participants (*n*)	Measurement method	Post-intervention	Follow-up			
						Week 4–7	3 months	6 months	1 year
Intensive-DLT (DLT, CDT)									
Abbasi [51]	DLT DLT + relaxation exercise^2^	3 *3*	16 *15*	Water displacement	47.5 (IQR 20.8)	62.4 (IQR 12.8)^3^			
Dayes^4^ [23]	DLT Phase-2 DLT	4 4	31 23	Manual circumference	29 ± 45^3^ 28 ± 25^3^		36 ± 35^3^ 38 ± 46^3^	45 ± 35^3^ 38 ± 34^3^	36 ± 64^3^ 37 ± 51^3^
Haghighat^4^ [52]	CDT	2–3	60	Water displacement	45.5 ± 13.3^5^				
Michopoulos [53]	CDT	4	12	Manual circumference	80.8 (79.1–105.0)^6^				
Gradalski [48]	DLT Bandaging^*2*^	2 *2*	25 *26*	Manual circumference	12.5^5,7^				
McNeely [21]	CB + MLD^8^ CB	4 4	8 10	Water displacement	56^9^ 47^9^				
McNeely [21]	CB + MLD^8^ CB	4 4	8 10	Manual circumference	62^9^ 44^9^				
MLD									
McNeely [21]	CB + MLD CB	4 4	8 10	Water displacement	56^9^ 47^9^				
McNeely [21]	CB + MLD CB	4 4	8 10	Manual circumference	62^9^ 44^9^				

^1^Assumes studies calculated relative reduction in EAV using comparable formulae, except for Gradalski who used different formula

^2^Data excluded as subgroup ineligible

^3^Intragroup difference *p* > .05

^4^Data provided by corresponding author

^5^No p value reported

^6^*p* < .05

^7^Calculated using formula for relative volume change described by Ancukiewicz et al. [55]

^8^Components of intensive-DLT

^9^Data calculated from published graph, *p* < .05

**Table 7:** Results for studies reporting pre- and post-intervention relative excess arm volume (%EAV)

Study	Intervention group	Participants (n)	Outcome	Measurement method	Pre-intervention %EAV^1^	Post-intervention %EAV^1^	Final reported %EAV^1^
Intensive-DLT (DLT, CDT)							
Dayes^2^ [23]	DLT Phase-2 DLT	31 23	%EAV	Manual circumference	23% ± 12 21% ± 7	16% ± 12^3^ 15% ± 9^3^	15% ± 15^3^ 15% ± 13^3^
Dayes^2^ [23]	DLT Phase-2 DLT	31 23	HRQOL	SF36 physical component	42.9 ± 7.5 44.6 ± 6.8	45.1 ± 7.3^4^ 44.8 ± 6.3^4^	43.7 ± 7.5^5^ 44.2 ± 7.0^5^
Dayes^2^ [23]	DLT Phase-2 DLT	31 23	HRQOL	SF36 mental component	43.7 ± 5.8 42.9 ± 6.0	43.9 ± 4.3^4^ 44.9 ± 6.4^4^	46.2 ± 6.2^5^ 43.6 ± 5.2^5^
Dayes^2^ [23]	DLT Phase-2 DLT	31 23	Function	DASH	29.9 ± 21.3 23.9 ± 19.9	31.2 ± 20.0^4^ 22.6 ± 19.1^4^	23.6 ± 17.2^5^ 20.5 ± 18.7^5^
Hwang [54]	CDT group1 CDT group2^4^	32 *25*	%EAV	Perometer	11% ± 5	10% ± 9^5^	14% ± 11^5^
Michoupolos [53]	DLT	12	%EAV	Manual circumference	28.8 (24.7–43.9)	5.4^5^ (‾2.0–9.0)	
Kim [49]	CDT + ARE CDT	20 20	HRQOL	SF36 physical component	68.25 ± 17.42 68.50 ± 11.01	85.12 ± 13.89^6^ 76.00 ± 12.73^6^	
Kim [49]	CDT + ARE CDT	20 20	HRQOL	SF36 mental component	66.25 ± 15.12 64.25 ± 16.85	75.25 ± 14.73^6^ 69.50 ± 17.63^6^	
CB							
Bahtiyarca^6^ [47]	CB + SLD CB	10 14	%EAV	Manual circumference	25.1 ± 11.3 24.7 ± 15.8	15.4 ± 9.8 14.4 ± 8.1	16.7 ± 11.1^7^ 17.8 ± 10.5^7^
Bahtiyarca^6^ [47]	CB + SLD CB	10 14	HRQOL	SF36 physical component	33.7 ± 11.0 39.6 ± 7.4	41.5 ± 9.4 48.1 ± 12.4	63.9 ± 17.6^6^ 57.9 ± 15.2^6^
Bahtiyarca^6^ [47]	CB + SLD CB	10 14	HRQOL	SF36 mental component	55.8 ± 10.9 52.2 ± 11.8	56.3 ± 9.2 63.3 ± 13.0	70.6 ± 14.9^6^ 68.1 ± 11.3^6^
Bahtiyarca^6^ [47]	CB + SLD CB	10 14	Function	QuickDASH	55.7 ± 16.2 54.4 ± 20.2	34.8 ± 17.5 35.7 ± 13.1	28.6 ± 9.7^6^ 27.7 ± 9.5^6^
Oh [50]	CB-spiral CB-spica	21 21	Function	DASH	32.8 ± 19.7 40.3 ± 11.7	29.5 ± 17.3^6^ 31.0 ± 13.9^6^	
Active resistive exercise (ARE)							
Kim [49]	CDT + ARE CDT	20 20	HRQOL	SF36 physical component	68.25 ± 17.42 68.50 ± 11.01	85.12 ± 13.89^6^ 76.00 ± 12.73^6^	
Kim [49]	CDT + ARE CDT	20 20	HRQOL	SF36 mental component	66.25 ± 15.12 64.25 ± 16.85	75.25 ± 14.73^6^ 69.50 ± 17.63^6^	

^1^Reported as mean ± SD or median (IQR) as recorded in the paper; %EAV = [swollen arm volume – non swollen arm volume]/non-swollen arm volume × 100

^2^Data provided by corresponding author

^3^Intergroup difference *p* > .05; 6-week findings reported, i.e. one week post-intervention

^4^Data excluded, group mean BCRL duration > 9 months

^5^No *p* values reported

^6^Study evaluated role of SLD but intervention group excluded whereas comparator (CB) eligible for review

^7^Intragroup difference at 6-month, *p* < .05

### Length of follow-up

Duration of follow-up ranged from 0 to 24 months (Table 5). The longest follow-up for a prospective trial was one year [23] and two years for a retrospective study [54]. Two trials completed follow-up at 6 weeks [49, 51] which is insufficient to determine the long-term benefit or retention of treatment effect. Four studies did not follow participants beyond completion of the study intervention period [21, 50, 52, 53].

## Findings

Tables 6 and 7 show findings, from the 10 studies according to intervention of interest and as published, including available* p* values, with additional findings for a subgroup of the study population provided by corresponding authors.

### Intensive-DLT/CDT

Two trials reported eligible data for both intervention and comparator groups. Dayes [23] reported no significant difference in between-group outcomes although 25% of those receiving intensive-DLT achieved ≥ 50%EAV reduction compared with 15% of those self-treating with hosiery. McNeely [21] reported greater reduction for those receiving CB + MLD (intensive-DLT) compared with CB alone, although they did not report the statistical significance and the study sample was smaller.

Dayes [23] and Michopoulos [53] reported pre- and post-intervention %EAV, categorised as pre-intervention moderate swelling (≥ 20% < 40%EAV) respectively reducing to mild (≥ 10% < 20%EAV) and minimal (< 10%EAV) post-intervention swelling. The reason for substantially greater 81% improvement (Table 6) reported by Michopoulos [53] is unknown as their baseline 29%EAV is similar to other studies. Only Hwang [54] reported no post-intervention reduction, although they considered lack of progression over the 2-year study period a positive outcome.

There were varied outcomes for HRQOL and function (Table 7). Kim [49] reported significant post-intervention HRQOL improvement for both groups (*p* < 0.05) with, of note, substantially higher pre-intervention SF-36 scores for both groups than Dayes [23] who reported no significant change for both groups. Gradalski [48] reported high satisfaction with treatment outcomes, self-rating 9.4/10, although it is unclear whether women rated satisfaction with the treatment experience or outcomes.

These limited findings suggest intensive-DLT can improve BCRL symptoms although, due to conflicted findings, evidence is insufficient to report benefit.

### Compression bandaging

Three studies reported demonstrated improvement with CB (Tables 6 and 7) although without shared or comparable outcomes or reporting methods [21, 47, 50]. These limited findings suggest CB can improve BCRL symptoms although, as the amount of improvement is unclear and no study compared CB with another intervention, evidence is insufficient to report benefit.

### Manual lymph drainage

One trial [21] tested the effect of MLD within the intensive-DLT package although statistical difference was not reported for the eligible subgroup of the population, so it is unclear whether these findings favour inclusion of MLD (Table 7). Although Gradalski [48] conducted a similar trial, the findings cannot be reported as the intervention group was ineligible for this review. One study addressed SLD [47] with improvements to volume, HRQOL and arm function found in both groups, but they concluded SLD added no benefit to CB (Table 7).

Therefore, evidence is insufficient to report benefit of MLD or SLD within the intensive-DLT package.

### Exercise

Kim [49] reported statistically significant improvement in post-intervention HRQOL for both groups receiving intensive-DLT (*p* < 0.05) although intergroup difference favoured the ARE intervention (*p* < 0.05) (Table 7). However, findings for reduction in arm size were excluded as reported using an ineligible method. There is insufficient evidence for the use of exercise to treat women with early BCRL.

### Summary of findings

Few comparisons could be made between studies reporting the same intervention or comparator due to inconsistencies and imprecision of results, small samples and lack of directly comparing treatments. There were serious limitations affecting some studies more than others, including lack of allocation concealment and analysis of completed assessments rather than by intention to treat per-protocol.

Evidence was lacking for long-term benefit of intensive-DLT for this population although there is weak evidence (grade B) that decongestive treatments, whether provided in the form of CDT or CB, with or without MLD, effectively reduces lymphedema in women with early BCRL (Table 8). This review could not determine the most effective combination of treatment elements to reduce EAV or improve patient-centred outcomes, or the optimum treatment duration for women with early BCRL.

**Table 8:** Summary of individual study findings for women with breast cancer–related lymphoedema (BCRL) symptom duration of less than 12 months

Study		*N*	EAV		HRQOL		Arm function		Arm heaviness		Patient benefit	Group difference, *p*^*1*^
CDT/CDPT/DLT												
Abbasi [51]	CDT^2^	16	+									n/a
Dayes^3^ [23]	DLT^2^ Hosiery^2^	31 24	+ +		O O		O O					> .05
Gradalski [48]	CDT^2,4^	25	+		+						+	> .05
Haghighat^3^ [52]	CDT^2^	60	+						NR			n/a
Hwang [54]	CDT	32	O									n/a
Michoupolos [53]	CDT	12	+ +									n/a
MLD												
McNeely [21]	CB + MLD^2^ CB^2^	8 10	+ +									NR
CB												
Bahtiyarca [47]	CB/SLD CB	10 14	+ +		+ +		+ +					
Oh [50]	Spiral method Spica method	21 21					+ +					> .05
Active resistive exercise (ARE)												
Kim [49]	CDPT + ARE CDPT	20 20			+ +							> .05

+  = improved, +  +  = substantially improved, O = unchanged, ? = data unclear, *NR* not reported, *n/a* no comparison group in review

^1^As reported in study

^2^Subgroup of women with BCRL duration < 12 months

^3^Data provided by corresponding author

^4^CB subgroup excluded

## Discussion

This review provides the only systematic examination of evidence for treatment of women with < 12-month BCRL duration, with no relevant studies published since review searches were updated in September 2022. Therefore, there is no evidence to support change in clinical practice by UK practitioners who routinely offer women self-treatment with hosiery or by practitioners in other countries offering initial intensive-DLT as standard care. The paucity of eligible papers and lack of trials recruiting only women with early BCRL highlight a big gap in research addressing management of early BCRL.

Lack of evidence for superiority of any treatment for women with BCRL has been previously reported [24–26, 44]. While Rangon et al. [25] suggested their review findings favoured intensive-DLT over other multimodal therapies, they included a smaller selection of trials which mostly compared variations of intensive-DLT, adding, removing or replacing DLT components. Dayes et al. [23] reported an unexpected finding of no significant difference in outcomes for women randomised to two-phase DLT or self-treatment with hosiery which was contrary to findings of an earlier trial by Badger et al. [19] which favoured CB over self-treatment using hosiery. The reason for these different findings is unknown although participants in the earlier trial [19] had no previous treatment experience, whereas an unknown number of women in Dayes trial [23] had previously received two-phase DLT or worn hosiery.

Although Hwang et al. [54] were the only study not to report improvement with intensive-DLT, they considered it meaningful that BCRL severity had not progressed over the 24-month follow-up period. Several studies highlight the importance of applying effective treatment at an early stage and monitoring symptom severity [56–59]. Two prospective surveillance studies reported progression of BCRL symptoms for 16% of women despite using hosiery for self-treatment [56, 57], although Blom et al. [56] reported worsened swelling for 57% of those not wearing compression garments. Bar Ad et al. [9] reported increased number of women progressing from mild to more severe BCRL over a 5-year review period. However, prospective surveillance studies do not address the treatment of established BCRL, including early BCRL, and no study addressed whether intensive-DLT can more effectively prevent or limit progression of BCRL symptoms. Further, the consequences of not appropriately treating established BCRL at an early stage are not considered.

This review also highlighted the paucity of research monitoring outcomes of importance to women. Few studies reported outcomes other than arm volume, although available evidence highlights the importance of monitoring symptom severity, such as arm heaviness, tightness and shoulder range of movement, and not just size [60, 61]. Women’s views of the value of treatment benefit and satisfaction with treatment were considered by only one study in this review [48], although the importance or meaningfulness for women and practitioners of the statistically significant findings reported by some studies in this review is unknown. Variation in measurement and reporting methods contributed to the paucity of outcome data, with minimal clinically important change for objective and self-report measures yet to be agreed, including arm volume, symptoms and HRQOL [10, 24, 26].

A strength of this review is the inclusion of literature from many databases with no date or language restriction. However, although recent expansion of bibliographic databases has index-linked many journals publishing lymphoedema papers, some relevant literature may have been missed by not searching all databases in 2022. In future updates, further consideration should be given to including other databases which could source additional eligible papers, for example, Embase may provide access to European studies that are not index-linked by Medline/PubMed. The impact of using single screening is unknown although we minimised the risk of missing studies at screening of title and abstract through careful use of EndNote, rechecking categories (EJ) and supervision by a senior experienced reviewer (DB). Heterogeneity of interventions and assessment methods, and variations in quality of study design and reporting, limited comparison of results and determination of the most effective treatment elements and optimal duration of treatment. Further, study samples were small, and few studies had sufficient post-intervention follow-up to address the chronicity of BCRL and potential for post-intervention treatment failure; these factors have been reported by multiple reviews of evidence for treatment of women with any BCRL duration [24, 62, 63]*.* Other limitations of this review highlight the paucity of research for this group, such as exclusion of findings reporting non-standard measurement methods and inclusion of one study group with mean ≤ 9-month BCRL duration while excluding the other with > 12-month duration. The lack of consensus regarding a standardised format for reporting and to inform comparisons between different protocols has been reported elsewhere [26, 64].

## Conclusion

The updated searches added four studies but highlight there is still no evidence to justify change to current practice for treatment of early BCRL, whether self-treatment with hosiery in the UK or two-phase DLT typically provided by other countries. As with the findings of the original report published in 2018 [35], there was weak evidence (grade B) [65] for the impact of DLT for women with early BCRL regardless of the combination of treatment components, and the heterogeneous treatment protocols did not permit conclusions to be drawn about optimal treatment components for initial BCRL treatment or the required duration to reduce EAV and improve patient-centred outcomes.

The findings of this review confirm that, four years after the original review [35], there is still a need for a definitive trial of treatment effectiveness for women with early BCRL. Researchers with an interest in this area need to design adequately powered studies which recruit only women with < 12-month BCRL duration and monitor treatment outcomes for at least one year and preferably five years or more. Collaborations should be encouraged between groups of lymphoedema researchers to create comparable treatment protocols, outcome measures and reporting methods. Further research is also required to determine measurement methods which are meaningful to women, including minimal clinically important changes for outcomes; the inclusion of women with BCRL in designing studies could help to identify priority outcomes and select appropriate patient reported outcome measures. The impact of intensive practitioner-delivered intensive-DLT and self-treatment with hosiery on progression of BCRL severity should also be addressed as prevention of symptom progression may be as important as improving symptoms.

### Supplementary Information

Below is the link to the electronic supplementary material.

Supplementary file1 (DOCX 594 KB)

## Data Availability

All data generated and/or analysed during this review are included in the published article and its supplementary files or in Jeffs et al. [35].
