# Supplementary material for: Evidence for the clinical effectiveness of decongestive lymphoedema treatment for breast cancer–related arm lymphoedema, a systematic review
Source: Support Care Cancer. 2024 Aug 2;32(8):568. doi: 10.1007/s00520-024-08759-x (PMC11296965; doi:10.1007/s00520-024-08759-x)
Supplement: Supplementary file 1 — Supplementary file1 (DOCX 594 KB) [file 520_2024_8759_MOESM1_ESM.docx]

## Online Resource 1: Preferred Reporting Item for Systematic reviews and Meta-Analysis (PRISMA) 2020 checklist

| ***Section and Topic*** | ***Item #*** | ***Checklist item*** | ***Reported on page #*** |
| --- | --- | --- | --- |
| **TITLE** | | |  |
| Title | 1 | Identify the report as a systematic review. | 1 |
| **ABSTRACT** | | |  |
| Abstract | 2 | See the PRISMA 2020 for Abstracts checklist. | 2 |
| **INTRODUCTION** | | |  |
| Rationale | 3 | Describe the rationale for the review in the context of existing knowledge. | 3-4 |
| Objectives | 4 | Provide an explicit statement of the objective(s) or question(s) the review addresses. | 4 |
| **METHODS** | | |  |
| Eligibility criteria | 5 | Specify the inclusion and exclusion criteria for the review and how studies were grouped for the syntheses. | 6 (Table 1) and 7 |
| Information sources | 6 | Specify all databases, registers, websites, organisations, reference lists and other sources searched or consulted to identify studies. Specify the date when each source was last searched or consulted. | 6-7, Online Resource 3 and 4 |
| Search strategy | 7 | Present the full search strategies for all databases, registers and websites, including any filters and limits used. | Online Resource 2 |
| Selection process | 8 | Specify the methods used to decide whether a study met the inclusion criteria of the review, including how many reviewers screened each record and each report retrieved, whether they worked independently, and if applicable, details of automation tools used in the process. | 5 (fig 1) and 7 |
| Data collection process | 9 | Specify the methods used to collect data from reports, including how many reviewers collected data from each report, whether they worked independently, any processes for obtaining or confirming data from study investigators, and if applicable, details of automation tools used in the process. | 5 (fig 1) and 7, Online Resource 6 |
| Data items | 10a | List and define all outcomes for which data were sought. Specify whether all results that were compatible with each outcome domain in each study were sought (e.g. for all measures, time points, analyses), and if not, the methods used to decide which results to collect. | 6 (Table 1), 7, and Online Resource 6 |

| ***Section and Topic*** | ***Item #*** | ***Checklist item*** | ***Reported on page #*** |
| --- | --- | --- | --- |
| Data items | 10b | List and define all other variables for which data were sought (e.g. participant and intervention characteristics, funding sources). Describe any assumptions made about any missing or unclear information. | Online Resources 5 and 6 |
| Study risk of bias assessment | 11 | Specify the methods used to assess risk of bias in the included studies, including details of the tool(s) used, how many reviewers assessed each study and whether they worked independently, and if applicable, details of automation tools used in the process. | 7 and 10 (Table 2a, 2b) |
| Effect measures | 12 | Specify for each outcome the effect measure(s) (e.g. risk ratio, mean difference) used in the synthesis or presentation of results. | n/a |
| Synthesis methods | 13a | Describe the processes used to decide which studies were eligible for each synthesis (e.g. tabulating the study intervention characteristics and comparing against the planned groups for each synthesis (item #5)). | n/a |
|  | 13b | Describe any methods required to prepare the data for presentation or synthesis, such as handling of missing summary statistics, or data conversions. | n/a |
|  | 13c | Describe any methods used to tabulate or visually display results of individual studies and syntheses. | 7 and 8 |
|  | 13d | Describe any methods used to synthesize results and provide a rationale for the choice(s). If meta-analysis was performed, describe the model(s), method(s) to identify the presence and extent of statistical heterogeneity, and software package(s) used. | n/a |
|  | 13e | Describe any methods used to explore possible causes of heterogeneity among study results (e.g. subgroup analysis, meta-regression). | n/a |
|  | 13f | Describe any sensitivity analyses conducted to assess robustness of the synthesized results. | n/a |
| Reporting bias assessment | 14 | Describe any methods used to assess risk of bias due to missing results in a synthesis (arising from reporting biases). | n/a |
| Certainty assessment | 15 | Describe any methods used to assess certainty (or confidence) in the body of evidence for an outcome. | n/a |
| Study selection | 16a | Describe the results of the search and selection process, from the number of records identified in the search to the number of studies included in the review, ideally using a flow diagram. | 8 and 9 (Figure 2) |
| Study selection | 16b | Cite studies that might appear to meet the inclusion criteria, but which were excluded, and explain why they were excluded. | 9 |

| ***Section and Topic*** | ***Item #*** | | ***Checklist item*** | | ***Reported on page #*** |
| --- | --- | --- | --- | --- | --- |
| **RESULTS** | | | | |  |
| Study characteristics | | 17 | | Cite each included study and present its characteristics. | 11-20, Table 3 (p12-16) |
| Risk of bias in studies | | 18 | | Present assessments of risk of bias for each included study. | 9-11, Table 2a and 2b (p10) |
| Results of individual studies | | 19 | | For all outcomes, present, for each study: (a) summary statistics for each group (where appropriate) and (b) an effect estimate and its precision (e.g. confidence/credible interval), ideally using structured tables or plots. | 21-26, Table 5a and 5b (p21-23) |
| Results of syntheses | | 20a | | For each synthesis, briefly summarise the characteristics and risk of bias among contributing studies. | n/a |
|  | | 20b | | Present results of all statistical syntheses conducted. If meta-analysis was done, present for each the summary estimate and its precision (e.g. confidence/credible interval) and measures of statistical heterogeneity. If comparing groups, describe the direction of the effect. | n/a |
|  | | 20c | | Present results of all investigations of possible causes of heterogeneity among study results. | n/a |
|  | | 20d | | Present results of all sensitivity analyses conducted to assess the robustness of the synthesized results. | n/a |
| Reporting biases | | 21 | | Present assessments of risk of bias due to missing results (arising from reporting biases) for each synthesis assessed. | n/a |
| Certainty of evidence | | 22 | | Present assessments of certainty (or confidence) in the body of evidence for each outcome assessed. | 26 and 27 (Table 6) |
| **DISCUSSION** | | | | |  |
| Discussion | 23a | | Provide a general interpretation of the results in the context of other evidence. | | 28-30 |
|  | 23b | | Discuss any limitations of the evidence included in the review. | | 29 |
|  | 23c | | Discuss any limitations of the review processes used. | | 29 |
|  | 23d | | Discuss implications of the results for practice, policy, and future research. | | 29-30 |

| ***Section and Topic*** | ***Item #*** | ***Checklist item*** | ***Reported on page #*** |
| --- | --- | --- | --- |
| **OTHER INFORMATION** | | |  |
| Registration and protocol | 24a | Provide registration information for the review, including register name and registration number, or state that the review was not registered. | 4 |
|  | 24b | Indicate where the review protocol can be accessed, or state that a protocol was not prepared. | 4 |
|  | 24c | Describe and explain any amendments to information provided at registration or in the protocol. | 6-7 |
| Support | 25 | Describe sources of financial or non-financial support for the review, and the role of the funders or sponsors in the review. | 1 |
| Competing interests | 26 | Declare any competing interests of review authors. | 1 |
| Availability of data, code and other materials | 27 | Report which of the following are publicly available and where they can be found: template data collection forms; data extracted from included studies; data used for all analyses; analytic code; any other materials used in the review. | Table 3 (p12-16), Online Resource 6 |

## Online Resource 2: Final search strategy

| Online Resource 2: Final search strategy | |
| --- | --- |
| 1. Lymphoedema OR lymphedema   OR lymphoedemas OR lymphedemas |  |
| 1. Breast AND (cancer OR carcinoma OR tumour OR tumor OR malignancy OR neoplasm) | in title, abstract, subject heading, keyword |
| 1. Mastectomy |  |
| 1. 2 OR 3 |  |
| 1. 1 AND 4 |  |
| 1. (lymphoedema OR lymphedema OR lymphoedemas OR lymphedemas) AND 'review' | in title only |
| 1. 5 OR 6. |  |

The following databases were searched on 6^th^ July 2016 (unless stated otherwise) to identify studies published in the commercial literature.

| Allied and Contemporary Medicine (AMED) | |
| --- | --- |
| Search number | Parameters |
| 1 | (lymphoedema OR lymphedema OR lymphoedemas OR lymphedemas).ti,ab |
| 2 | ((Breast cancer) OR (breast malignancy) OR (breast tumour) OR (breast tumor) OR (breast carcinoma) OR (breast neoplasm) OR mastectomy).ti,ab |
| 3 | exp BREAST NEOPLASMS/ |
| 4 | exp LYMPHEDEMA/ |
| 5 | ((lymphoedema OR lymphedema OR lymphoedemas OR lymphedemas) AND review).ti |
| 6 | 1 OR 4 |
| 7 | 2 OR 3 |
| 8 | 6 AND 7 |
| 9 | 5 OR 8 |

| Biomed Central | |
| --- | --- |
| Search number | Parameters (searched on 1^st^ July 2015, but could not be replicated in 2016) |
| 1 | (((cancer OR malignancy OR tumour OR tumor OR neoplasm OR carcinoma[TW]) AND (breast[TW])) AND (lymphoedema OR lymphedema OR lymphoedemas OR lymphedemas[TW])) OR (mastectomy[TW] AND (lymphoedema OR lymphedema OR lymphoedemas OR lymphedemas[TW])) OR ((lymphoedema OR lymphedema OR lymphoedemas OR lymphedemas[TW]) AND (review[TW])) |

| BIOSIS | |
| --- | --- |
| Search number | Parameters (for period to 2008) |
| 1 | TS=(cancer OR malignancy OR tumour OR tumor OR neoplasm OR carcinoma); *Indexes=BCI Timespan=All years* |
| 2 | TS=breast; *Indexes=BCI Timespan=All years* |
| 3 | #2 AND #1; *Indexes=BCI Timespan=All years* |
| 4 | TS=mastectomy; *Indexes=BCI Timespan=All years* |
| 5 | #4 OR #3; *Indexes=BCI Timespan=All years* |
| 6 | TS=(lymphoedema OR lymphedema OR lymphoedemas OR lymphedemas); *Indexes=BCI Timespan=All years* |
| 7 | #6 AND #5; *Indexes=BCI Timespan=All years* |
| 8 | TI=(cancer OR malignancy OR tumour OR tumor OR neoplasm OR carcinoma); *Indexes=BCI Timespan=All years* |
| 9 | TI=breast; *Indexes=BCI Timespan=All years* |
| 10 | #9 AND #8; *Indexes=BCI Timespan=All years* |
| 11 | TI=mastectomy; *Indexes=BCI Timespan=All years* |
| 12 | #11 OR #10; *Indexes=BCI Timespan=All years* |
| 13 | TI=(lymphoedema OR lymphedema OR lymphoedemas OR lymphedemas); *Indexes=BCI Timespan=All years* |
| 14 | #13 AND #12; *Indexes=BCI Timespan=All years* |
| 15 | #14 OR #7; *Indexes=BCI Timespan=All years* |
| 16 | TI=review; *Indexes=BCI Timespan=All years* |
| 17 | #16 AND #13; *Indexes=BCI Timespan=All years* |
| 18 | #17 OR #15; *Indexes=BCI Timespan=All years* |

| British Nursing Index | |
| --- | --- |
| Search number | Parameters |
| 1 | (lymphoedema OR lymphedema OR lymphoedemas OR lymphedemas).ti,ab |
| 2 | ((Breast cancer) OR (breast malignancy) OR (breast tumour) OR (breast tumor) OR (breast carcinoma) OR (breast neoplasm) OR mastectomy).ti,ab |
| 3 | exp LYMPHOEDEMA/ |
| 4 | exp BREAST CANCER/ |
| 5 | ((lymphoedema OR lymphedema OR lymphoedemas OR lymphedemas) AND review).ti |
| 6 | 1 OR 3 |
| 7 | 2 OR 4 |
| 8 | 6 AND 7 |
| 9 | 5 OR 8 |

| CINAHL | |
| --- | --- |
| Search number | Parameters |
| 1 | (lymphoedema OR lymphedema OR lymphoedemas OR lymphedemas).ti,ab |
| 2 | ((Breast cancer) OR (breast malignancy) OR (breast tumour) OR (breast tumor) OR (breast carcinoma) OR (breast neoplasm) OR mastectomy).ti,ab |
| 3 | exp LYMPHEDEMA/ |
| 4 | exp BREAST NEOPLASMS/ |
| 5 | ((lymphoedema OR lymphedema OR lymphoedemas OR lymphedemas) AND review).ti |
| 6 | 1 OR 3 |
| 7 | 2 OR 4 |
| 8 | 6 AND 7 |
| 9 | 5 OR 8 |

| Cochrane Library | |
| --- | --- |
| Search number | Parameters |
| 1 | MeSH descriptor: [Lymphedema] explode all trees |
| 2 | MeSH descriptor: [Breast Neoplasms] explode all trees |
| 3 | cancer or malignancy or carcinoma or tumour or tumor or neoplasm:ti,ab,kw (Word variations have been searched) |
| 4 | breast:ti,ab,kw (Word variations have been searched) |
| 5 | mastectomy:ti,ab,kw (Word variations have been searched) |
| 6 | lymphoedema or lymphedema or lymphoedemas or  lymphedemas:ti,ab,kw (Word variations have been searched) |
| 7 | #1 or #6 |
| 8 | #3 and #4 |
| 9 | #8 or #2 or #5 |
| 10 | #7 and #9 |
| 11 | review:ti (Word variations have been searched) |
| 12 | #1 and #11 |
| 13 | #10 or #12 |

| Embase | |
| --- | --- |
| Search number | Parameters |
| 1 | exp lymphedema/ |
| 2 | lymph*edema?.mp. [mp=title, abstract, heading word, drug trade name, original title, device manufacturer, drug manufacturer, device trade name, keyword] |
| 3 | 1 or 2 |
| 4 | exp breast cancer/ |
| 5 | exp breast tumor/ |
| 6 | exp breast carcinoma/ |
| 7 | (breast adj3 cancer).mp. [mp=title, abstract, heading word, drug trade name, original title, device manufacturer, drug manufacturer, device trade name, keyword] |
| 8 | (breast adj3 tumo*r).mp. [mp=title, abstract, heading word, drug trade name, original title, device manufacturer, drug manufacturer, device trade name, keyword] |
| 9 | (breast adj3 malignancy).mp. [mp=title, abstract, heading word, drug trade name, original title, device manufacturer, drug manufacturer, device trade name, keyword] |
| 10 | (breast adj3 carcinoma).mp. [mp=title, abstract, heading word, drug trade name, original title, device manufacturer, drug manufacturer, device trade name, keyword] |
| 11 | (breast adj3 neoplasm).mp. [mp=title, abstract, heading word, drug trade name, original title, device manufacturer, drug manufacturer, device trade name, keyword] |
| 12 | mastectomy.mp. |
| 13 | (lymph*edema? and review).ti. |
| 14 | 4 or 5 or 6 or 7 or 8 or 9 or 10 or 11 or 12 |
| 15 | 3 and 14 |
| 16 | 13 or 15 |

| HMIC | |
| --- | --- |
| Search number | Parameters |
| 1 | (breast adj3 cancer).mp. [mp=title, other title, abstract, heading words] |
| 2 | (breast adj3 tumo*r).mp. [mp=title, other title, abstract, heading words] |
| 3 | (breast adj3 malignancy).mp. [mp=title, other title, abstract, heading words] |
| 4 | (breast adj3 carcinoma).mp. [mp=title, other title, abstract, heading words] |
| 5 | (breast adj3 neoplasm).mp. [mp=title, other title, abstract, heading words] |
| 6 | mastectomy.mp. |
| 7 | exp Breast cancer/ |
| 8 | 1 or 2 or 3 or 4 or 5 or 6 or 7 |
| 9 | lymph*edema?.mp. |
| 10 | 8 and 9 |
| 11 | (lymph*edema? and review).ti. |
| 12 | 10 or 11 |

| Medline | |
| --- | --- |
| Search number | Parameters |
| 1 | exp Breast Neoplasms/ |
| 2 | (breast adj3 cancer).mp. [mp=title, abstract, original title, name of substance word, subject heading word, keyword heading word, protocol supplementary concept word, rare disease supplementary concept word, unique identifier] |
| 3 | (breast adj3 tumo*r).mp. [mp=title, abstract, original title, name of substance word, subject heading word, keyword heading word, protocol supplementary concept word, rare disease supplementary concept word, unique identifier] |
| 4 | (breast adj3 malignancy).mp. [mp=title, abstract, original title, name of substance word, subject heading word, keyword heading word, protocol supplementary concept word, rare disease supplementary concept word, unique identifier] |
| 5 | (breast adj3 carcinoma).mp. [mp=title, abstract, original title, name of substance word, subject heading word, keyword heading word, protocol supplementary concept word, rare disease supplementary concept word, unique identifier] |
| 6 | (breast adj3 neoplasm).mp. [mp=title, abstract, original title, name of substance word, subject heading word, keyword heading word, protocol supplementary concept word, rare disease supplementary concept word, unique identifier] |
| 7 | mastectomy.mp. |
| 8 | 1 or 2 or 3 or 4 or 5 or 6 or 7 |
| 9 | exp Lymphedema/ |
| 10 | lymph*edema?.mp. [mp=title, abstract, original title, name of substance word, subject heading word, keyword heading word, protocol supplementary concept word, rare disease supplementary concept word, unique identifier] |
| 11 | 9 or 10 |
| 12 | 8 and 11 |
| 13 | (lymph*edema? and review).ti. |
| 14 | 12 or 13 |

| Physiotherapy Evidence database (PEDro) | |
| --- | --- |
| Search number | Parameters |
| 1 | breast AND *edema |

| PsycARTICLES | |
| --- | --- |
| Search number | Parameters |
| 1 | (breast adj3 cancer).mp. [mp=title, abstract, full text, caption text] |
| 2 | (breast adj3 tumo*r).mp. [mp=title, abstract, full text, caption text] |
| 3 | (breast adj3 malignancy).mp. [mp=title, abstract, full text, caption text] |
| 4 | (breast adj3 carcinoma).mp. [mp=title, abstract, full text, caption text] |
| 5 | (breast adj3 neoplasm).mp. [mp=title, abstract, full text, caption text] |
| 6 | mastectomy.mp. |
| 7 | lymph*edema?.mp. [mp=title, abstract, full text, caption text] |
| 8 | (lymph*edema? and review).ti. |
| 9 | 1 or 2 or 3 or 4 or 5 or 6 |
| 10 | 7 and 9 |
| 11 | 8 or 10 |

| PsycINFO | |
| --- | --- |
| Search number | Parameters |
| 1 | exp Breast Neoplasms/ |
| 2 | (breast adj3 cancer).mp. [mp=title, abstract, heading word, table of contents, key concepts, original title, tests & measures] |
| 3 | (breast adj3 tumo*r).mp. [mp=title, abstract, heading word, table of contents, key concepts, original title, tests & measures] |
| 4 | (breast adj3 malignancy).mp. [mp=title, abstract, heading word, table of contents, key concepts, original title, tests & measures] |
| 5 | (breast adj3 carcinoma).mp. [mp=title, abstract, heading word, table of contents, key concepts, original title, tests & measures] |
| 6 | (breast adj3 neoplasm).mp. [mp=title, abstract, heading word, table of contents, key concepts, original title, tests & measures] |
| 7 | mastectomy.mp. |
| 8 | 1 or 2 or 3 or 4 or 5 or 6 or 7 |
| 9 | lymph*edema?.mp. [mp=title, abstract, heading word, table of contents, key concepts, original title, tests & measures] |
| 10 | lymph*edema?.ti. |
| 11 | review.ti. |
| 12 | 8 and 9 |
| 13 | 10 and 11 |
| 14 | 12 or 13 |

| PubMed | |
| --- | --- |
| Search number | Parameters |
| 1 | Search mastectomy[Title] |
| 2 | Search (breast cancer[MeSH Terms]) OR mastectomies[MeSH Terms] |
| 3 | Search (breast[Title/Abstract]) AND (neoplasm[Title/Abstract] OR neoplasms[Title/Abstract] OR carcinoma[Title/Abstract] OR cancer[Title/Abstract] OR tumour[Title/Abstract] OR tumor[Title/Abstract] OR malignancy[Title/Abstract] OR malignancies[Title/Abstract]) |
| 4 | Search (((lymphedema[Title/Abstract] OR lymphedemas[Title/Abstract] OR lymphoedema[Title/Abstract] OR lymphoedemas[Title/Abstract])) OR lymphedema[MeSH Terms]) OR lymphedemas[MeSH Terms] |
| 5 | Search (review[Title]) AND ((((lymphedema[Title/Abstract] OR lymphedemas[Title/Abstract] OR lymphoedema[Title/Abstract] OR lymphoedemas[Title/Abstract])) OR lymphedema[MeSH Terms]) OR lymphedemas[MeSH Terms]) |
| 6 | Search ((((breast[Title/Abstract]) AND (neoplasm[Title/Abstract] OR neoplasms[Title/Abstract] OR carcinoma[Title/Abstract] OR cancer[Title/Abstract] OR tumour[Title/Abstract] OR tumor[Title/Abstract] OR malignancy[Title/Abstract] OR malignancies[Title/Abstract]))) OR ((breast cancer[MeSH Terms]) OR mastectomies[MeSH Terms])) OR mastectomy[Title] |
| 7 | Search ((((((breast[Title/Abstract]) AND (neoplasm[Title/Abstract] OR neoplasms[Title/Abstract] OR carcinoma[Title/Abstract] OR cancer[Title/Abstract] OR tumour[Title/Abstract] OR tumor[Title/Abstract] OR malignancy[Title/Abstract] OR malignancies[Title/Abstract]))) OR ((breast cancer[MeSH Terms]) OR mastectomies[MeSH Terms])) OR mastectomy[Title])) AND ((((lymphedema[Title/Abstract] OR lymphedemas[Title/Abstract] OR lymphoedema[Title/Abstract] OR lymphoedemas[Title/Abstract])) OR lymphedema[MeSH Terms]) OR lymphedemas[MeSH Terms]) |
| 8 | Search ((((((mastectomy[Title]) OR ((breast cancer[MeSH Terms]) OR mastectomies[MeSH Terms])) OR ((breast[Title/Abstract]) AND (neoplasm[Title/Abstract] OR neoplasms[Title/Abstract] OR cancer[Title/Abstract] OR carcinoma[Title/Abstract] OR tumour[Title/Abstract] OR tumor[Title/Abstract] OR malignancy[Title/Abstract])))) AND ((((lymphedema[Title/Abstract] OR lymphoedema[Title/Abstract] OR lymphedemas[Title/Abstract] OR lymphoedemas[Title/Abstract])) OR lymphedema[MeSH Terms]) OR lymphedemas[MeSH Terms]))) OR ((review[Title]) AND ((((lymphedema[Title/Abstract] OR lymphoedema[Title/Abstract] OR lymphedemas[Title/Abstract] OR lymphoedemas[Title/Abstract])) OR lymphedema[MeSH Terms]) OR lymphedemas[MeSH Terms])) |

| Scopus | |
| --- | --- |
| Search number | Parameters |
| 1 | (((TITLE-ABS-KEY(cancer OR malignancy OR tumour OR tumor OR neoplasm OR carcinoma) AND TITLE-ABS-KEY(Breast)) OR TITLE-ABS-KEY(Mastectomy)) AND TITLE-ABS-KEY(lymphoedema OR lymphedema OR lymphoedemas OR lymphedemas)) OR (TITLE(lymphoedema OR lymphedema OR lymphoedemas OR lymphedemas) AND TITLE(Review)) |

| Turning Research into Practice (TRIP) | |
| --- | --- |
| Search number | Parameters (searched on 1^st^ July 2015) |
| 1 | ((((title:cancer or malignancy or tumour or tumor or neoplasm or carcinoma) and (title:breast)) or (title:mastectomy)) and (title:lymphoedema or lymphedema)) or ((title:lymphoedema or lymphedema) and (title:review)) |

| Web of Science | |
| --- | --- |
| Search number | Parameters |
| 1 | TS=mastectomy OR TI=mastectomy  Indexes=SCI-EXPANDED, SSCI, A&HCI, CPCI-S, CPCI-SSH, ESCI Timespan=1900-2016 |
| 2 | TI=(breast cancer) OR TI=(breast malignancy) OR TI=(breast tumour) OR TI=(breast tumor) OR TI=(breast neoplasm) OR TI=(breast carcinoma) |
| 3 | TS=(breast cancer) OR TS=(breast malignancy) OR TS=(breast tumour) OR TS=(breast tumor) OR TS=(breast neoplasm) OR TS=(breast carcinoma) |
| 4 | #3 OR #2 OR #1 |
| 5 | TS=(lymphoedema OR lymphedema OR lymphoedemas OR lymphedemas) |
| 6 | TI=(lymphoedema OR lymphedema OR lymphoedemas OR lymphedemas) |
| 7 | #6 OR #5 |
| 8 | #7 AND #4 |
| 9 | TI=review |
| 10 | #9 AND #6 |
| 11 | #10 OR #8 |

| WorldCat | |
| --- | --- |
| Search number | Parameters |
| 1 | ((kw: lymphedema or kw: lymphoedema or kw: lymphoedemas or kw: lymphedemas) and kw: mastectomy) or ((kw: lymphedema or kw: lymphoedema or kw: lymphoedemas or kw: lymphedemas) and (kw: breast and (kw: cancer OR kw: malignancy OR kw: tumour OR kw: tumor OR kw: neoplasm OR kw: carcinoma))) or ((kw: lymphoedema or kw: lymphedema or kw: lymphoedemas or kw: lymphedemas) and ti: review) |

The following databases were searched on 4^th^ July 2016 to identify studies conducted but not published in the commercial literature (i.e. gray literature):

| ClinicalTrials.gov (USA) | |
| --- | --- |
| Search number | Parameters |
| 1 | “breast cancer” AND lymphedema |

| Controlled Trials Register (ISRCTN) | |
| --- | --- |
| Search number | Parameters |
| 1 | Condition: breast cancer |
| 2 | Text search: lymphoedema OR lymphedema |

| Gray Literature Report: www.greylit.org/ | |
| --- | --- |
| Search number | Parameters |
| 1 | Lymphoedema |
| 2 | Lymphedema |

| Gray Literature Report: [www.opengrey.eu](http://www.opengrey.eu) | |
| --- | --- |
| Search number | Parameters |
| 1 | Lymphoedema |
| 2 | Lymphedema |

| International Clinical Trials Registry Platform (ICTRP) | |
| --- | --- |
| Search number | Parameters |
| 1 | Breast cancer AND lymphedema |

| WorldCat ArticleFirst (OCLC) | |
| --- | --- |
| Search number | Parameters |
| 1 | ((kw: lymphedema or kw: lymphoedema or kw: lymphoedemas or kw: lymphedemas) and kw: mastectomy) or ((kw: lymphedema or kw: lymphoedema or kw: lymphoedemas or kw: lymphedemas) and (kw: breast and (kw: cancer OR kw: malignancy OR kw: tumour OR kw: tumor OR kw: neoplasm OR kw: carcinoma))) or ((kw: lymphoedema or kw: lymphedema or kw: lymphoedemas or kw: lymphedemas) and ti: review) |

| WorldCat Dissertations | |
| --- | --- |
| Search number | Parameters |
| 1 | ((kw: lymphedema or kw: lymphoedema or kw: lymphoedemas or kw: lymphedemas) and kw: mastectomy) or ((kw: lymphedema or kw: lymphoedema or kw: lymphoedemas or kw: lymphedemas) and (kw: breast and (kw: cancer OR kw: malignancy OR kw: tumour OR kw: tumor OR kw: neoplasm OR kw: carcinoma))) or ((kw: lymphoedema or kw: lymphedema or kw: lymphoedemas or kw: lymphedemas) and ti: review) |

| WorldCat PapersFirst | |
| --- | --- |
| Search number | Parameters |
| 1 | ((kw: lymphedema or kw: lymphoedema or kw: lymphoedemas or kw: lymphedemas) and kw: mastectomy) or ((kw: lymphedema or kw: lymphoedema or kw: lymphoedemas or kw: lymphedemas) and (kw: breast and (kw: cancer OR kw: malignancy OR kw: tumour OR kw: tumor OR kw: neoplasm OR kw: carcinoma))) or ((kw: lymphoedema or kw: lymphedema or kw: lymphoedemas or kw: lymphedemas) and ti: review) |

| WorldCat ProceedingsFirst | |
| --- | --- |
| Search number | Parameters |
| 1 | ((kw: lymphedema or kw: lymphoedema or kw: lymphoedemas or kw: lymphedemas) and kw: mastectomy) or ((kw: lymphedema or kw: lymphoedema or kw: lymphoedemas or kw: lymphedemas) and (kw: breast and (kw: cancer OR kw: malignancy OR kw: tumour OR kw: tumor OR kw: neoplasm OR kw: carcinoma))) or ((kw: lymphoedema or kw: lymphedema or kw: lymphoedemas or kw: lymphedemas) and ti: review) |

## Online Resource 3: Bibliographic databases searched, 2016, 2018, 2022

| Searches conducted on 4^th^ and 6^th^ July 2016 | | | |
| --- | --- | --- | --- |
| **Included in final search of commercial (black) literature** | **Included in final search of grey literature** | **Included in study protocol but excluded from final search** | **Excluded from protocol and final search of commercial & grey literature^1^** |
| Allied & Contemporary Medicine (AMED), Biomed Central^2^, BIOSIS Citation Index^3^, British Nursing Index, Cumulative Index to Nursing and Allied Health Literature (CINAHL), Cochrane Library, Embase, Health Management Information Consortium (HMIC), Medline, Physiotherapy Evidence database (PEDro), PsycARTICLES, PsycINFO, PubMed, Scopus, Turning Research into Practice (TRIP)^4^, Web of Science, WorldCat | ClinicalTrials.gov (USA), Controlled Trials Register, Grey Literature Report (http://www.greylit.org/ & www.opengrey.eu), International Clinical Trials Registry Platform (ICTRP), WorldCat ArticleFirst, WorldCat Dissertations, WorldCat PapersFirst, WorldCat ProceedingsFirst | PROSPERO, Centre for Reviews & Dissemination^5^; DARE^6^; Clinical Trials Registers/ UK Clinical Research Network (UKCRN)^7^; Conference Proceedings Citations Index (Web of Science)^8^; Evidence NHS UK (NICE)^9^; National Guideline Clearing House (USA)^5^ | African Journals Online, Airiti Library, Bielefeld Academic Search Engine (BASE), CiNii Articles, Europe PubMed Central, HubMed, Indian Citation Index, Latin American and Caribbean Health Sciences (LILACS), Russian Science Citation Index, Scientific Electronic Library Online (SciELO), American College of Physicians Journal Club, Amicus Canadian National Catalogue, British Library E-thesis online service (EThOS), DART-Europe E-theses Portal, TROVE National Library of Australia |
| *^1^Databases could not apply search strategy or added no new references*  *^2^Search completed to 1^st^ July 2015; original search strategy could not be replicated in July 2016*  *^3^Records only available for period 1969 to 2008 due to subscription limitations*  *^4^Search completed to 1^st^ July 2015; records obtained in July 2016 could not be accessed*  *^5^Not source for primary research as reports protocols for systematic reviews or summaries of evidence*  *^6^Database no longer maintained; DARE records included within Cochrane Library*  *^7^Now the Central Portfolio Management System; searches two databases already included in strategy*  *^8^Included within search of Web of Science Core Collection*  *^9^Searches range of databases already included in search strategy* | | | |

| Updated searches conducted on 6^th^ July 2018 | | | |
| --- | --- | --- | --- |
| **Included in updated search of commercial (black) literature** | **Included in updated search of grey literature** | **Included in hand-search of lymphoedema-specific journals** | **Included in original searches but not updated, with reasons specified** |
| Cumulative Index to Nursing and Allied Health Literature (CINAHL), Cochrane Library, Embase, Health Management Information Consortium (HMIC), Medline, Physiotherapy Evidence database (PEDro), PsycARTICLES, PsycINFO, PubMed, Scopus, Web of Science, WorldCat | None | European Journal of Lymphology; Journal of Lymphoedema; Journal of Phlebology and Lymphology; Lymphology | Allied and Contemporary Medicine (AMED)^1^, Biomed Central^2^, BIOSIS Citation Index^3^, British Nursing Index^1^, Turning Research into Practice (TRIP)^4^  Grey literature not updated:^5^  ClinicalTrials.gov (USA), Controlled Trials Register, Grey Literature Report (http://www.greylit.org/ & www.opengrey.eu), International Clinical Trials Registry Platform (ICTRP), WorldCat ArticleFirst (OCLC), WorldCat Dissertations, WorldCat PapersFirst, WorldCat ProceedingsFirst |
| *^1^Search completed to 6^th^ July 2016; could not be repeated in 2018 due to subscription limitations*  *^2^Search completed to 1^st^ July 2015; could not be repeated in 2016/2018 due to changes to Biomed Central*  *^3^Records only available for period 1969 to 2008 due to subscription limitations*  *^4^Search completed to 1^st^ July 2015; records obtained in 2016/2018 could not be accessed without additional subscription*  *^5^Findings not included in review, so no updated searches required* | | | |

| Updated searches conducted on 8^th^ September 2022 | | | |
| --- | --- | --- | --- |
| **Included in updated search of commercial (black) literature** | **Included in updated search of grey literature** | **Included in hand-search of lymphoedema-specific journals** | **Included in 6^th^ July 2018 search but not updated in 2022^1^** |
| Allied and Contemporary Medicine (AMED), Cumulative Index to Nursing and Allied Health Literature (CINAHL), PubMed | None | European Journal of Lymphology; Journal of Lymphoedema; Journal of Phlebology and Lymphology; Lymphology | Cochrane Library, Embase, Health Management Information Consortium (HMIC), Medline, Physiotherapy Evidence database (PEDro), PsycARTICLES, PsycINFO, Scopus, Web of Science, WorldCat |
| *^1^Findings from these databases did not add unique records to those included for full screening in 2018, so no need to update these searches in 2022* | | | |

## Online Resource 4: Characteristics of online lymphoedema-specific journals hand-searched in 2016, 2018 and 2022

| ***Hand-searches conducted on 6th July 2016, 6^th^ July 2018 and 8^th^ September 2022*** | | | | |
| --- | --- | --- | --- | --- |
| **Journal** | **Publisher** | **Focus** | **Dates/volumes searched** | **Volumes not searched** |
| European Journal of Lymphology^1^ | European Society of Lymphology; published since 1990 in French and/or English | Multidisciplinary focus on analysis, control and treatment of lymphatic diseases | 1990-1996, 2002-2017; volume 1(1) to 6, conference special, and 10(37) to 29(77) | numbers 3, 4, 7-8, 18, 21-36, 38, 39, 45, 47, 55, 57-62, 71, 75 |
| Journal of Lymphoedema^2^ | Wounds International, UK; published since 2006 in English | Peer-reviewed lymphoedema management resource; open-access website for clinicians | 2006-2018, volumes 1-13 | - |
| Journal of Phlebology and Lymphology^3^ | Brazilian Society of Phlebology and Lymphology; published since 2008 in English | Open access online journal of research and reviews for Latin America and the Caribbean | Volumes 1-11 (2008-2018) | - |
| Lymphology^4^ | International Society of Lymphology; published since 1968 in English | Clinical and basic science studies of lymphatic system and lymphoedema treatment; older papers open access | 1989-2016, volumes 22-49 | Volumes 1-21 (1968-1988) not available online; volumes 50 and 51 (2017 & 2018) were subscriber-only access |
| Lymphatic Research & Biology^5^ | Mary Ann Liebert Publications |  | Random 10% search |  |
| *^1^ www.eurolymphology.org/*  *^2^ www.journaloflymphoedema.com*  *^3^ www.pulsus.com/journal-phlebology-lymphology.html*  *^4^ www.journals.uair.arizona.edu/index.php/lymph/index*  *^5^ 8th September 2022 search only, www.liebertpub.com/loi/LRB* | | | | |

## Online Resource 5: Definitions list to support critical appraisal process

| ***Treatment modality*** | ***What should be included*** | |
| --- | --- | --- |
| Who provides treatment | - Lymphoedema therapist, whether HCP or MLD therapist, or other   May include school of treatment they follow, eg Casley-Smith, Foldi, Leduc. Indicates they have some training in Lymphoedema management   - OR patient self-management. | |
| Phase of treatment | - Intensive or phase 1 - Maintenance/patient self-management or phase 2 | |
| Duration of treatment | - Patient self-management phase 2 treatment is generally accepted to be daily self-care on a continuous basis. They are likely only to mention duration if an additional element is added, e.g. weekly exercise class. - Phase 1 intensive treatment is usually for 3-8 weeks, so should specify sessions per week and number of weeks treatment OR number of sessions over x weeks | |
| Compression bandaging | - Type (e.g. low stretch/cohesive bandage) or brand (e.g., Rosidal, Coban2) - Frequency applied? e.g. daily, 2-3x/wk - Applied by? E.g. therapist, patient - Padding used? for comfort or also to focus pressure? - Are bandages in place overnight and over weekend? | |
| Manual Lymphatic Drainage (MLD) | - Applied by? If patient, may be referred to as SLD (self/simple lymphatic drainage) - How many sessions per week - Duration of session - Method used, e.g. Casley-Smith, Foldi, Leduc | |
| Intermittent Pneumatic Compression Therapy (IPC/PCT) | - Type of pump used (intermittent, sequential) – may state make - Number of chambers (3,5,10, etc) - Pressure used (mm Hg) - Duration of session (mins) - Frequency of session / week | |
| LLLT (laser treatment) | - Type of laser used (usually state model) - Wavelength used - Output: power (mW) and pulse frequency (Hz) - Dose (J/cm2) - Duration of session (mins) - Number and location of sites used - Patient position for treatment - Frequency of session / week | |
| Exercise | - What done – description of exercises or muscles worked, number of reps - How often - Whether hosiery/bandages worn during exercise | |
| Volume measurement | - Can use Perometry, tape /girth measure or water displacement – must describe & reference method - Describe how excess limb volume (ELV) is calculated (eg Kunke) - Must measure non-swollen limb and use the difference between 2 limbs (ELV) and compare this with the normal limb as reference | Calcs should look something like this:  ELV = swollen - non-swollen limb  Relative change = ELV t1 – ELV t0  Percentage volume reduction (PVR)  = (ELV t1 – ELV t0) x100  ELV t0 |
| Bioimpedance  (BIS) | - Should state which machine used - May report either as either an impedance ratio, or an L-Dex ratio | |
| Tissue Dielectric constant | - Should state depth achieved by probe - Measurement reported in relative units, between 1-80 | |

## Online Resource 6: Data extraction form

**(**content modified from JBI-MAStARI data extraction tool)


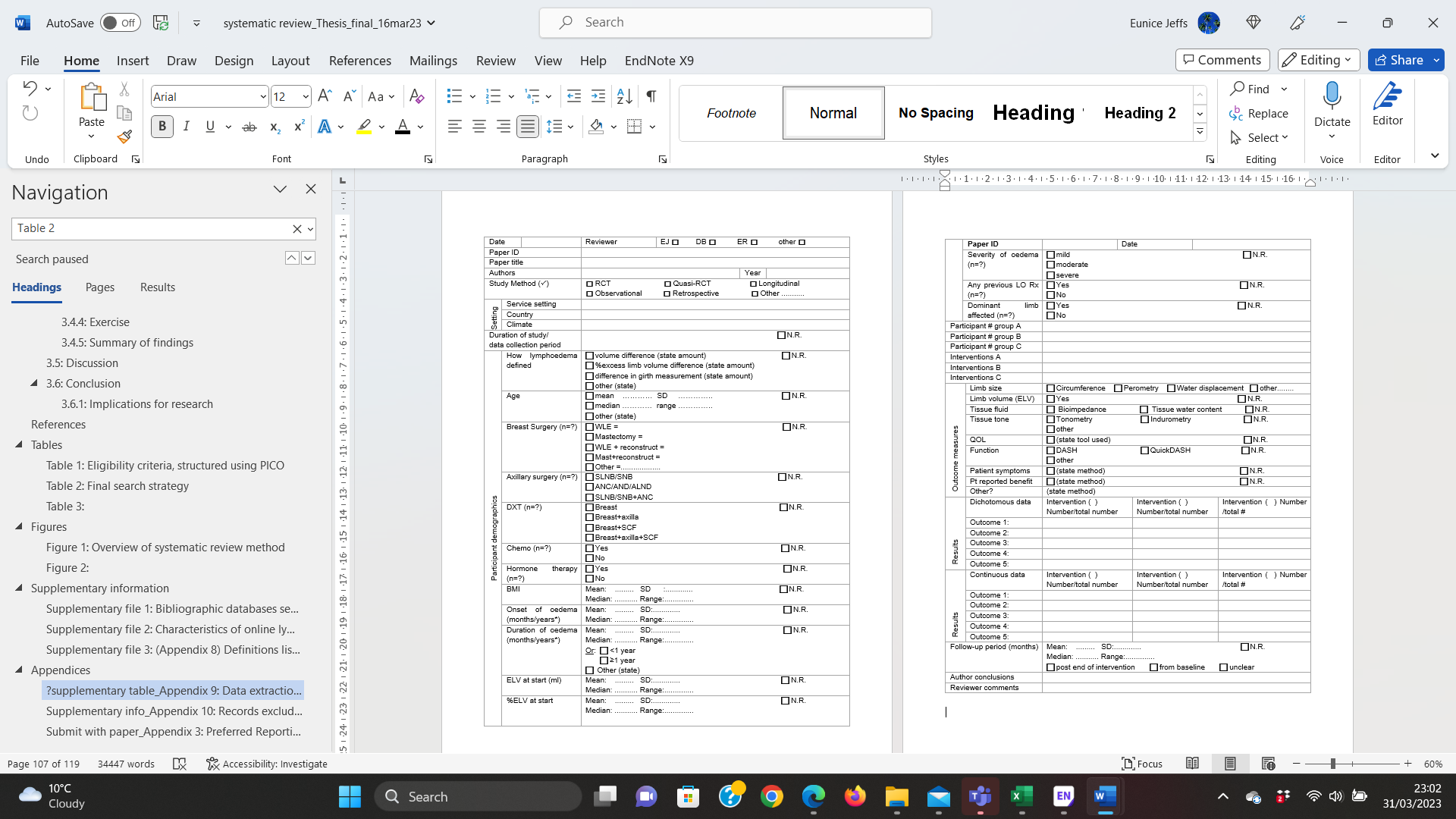


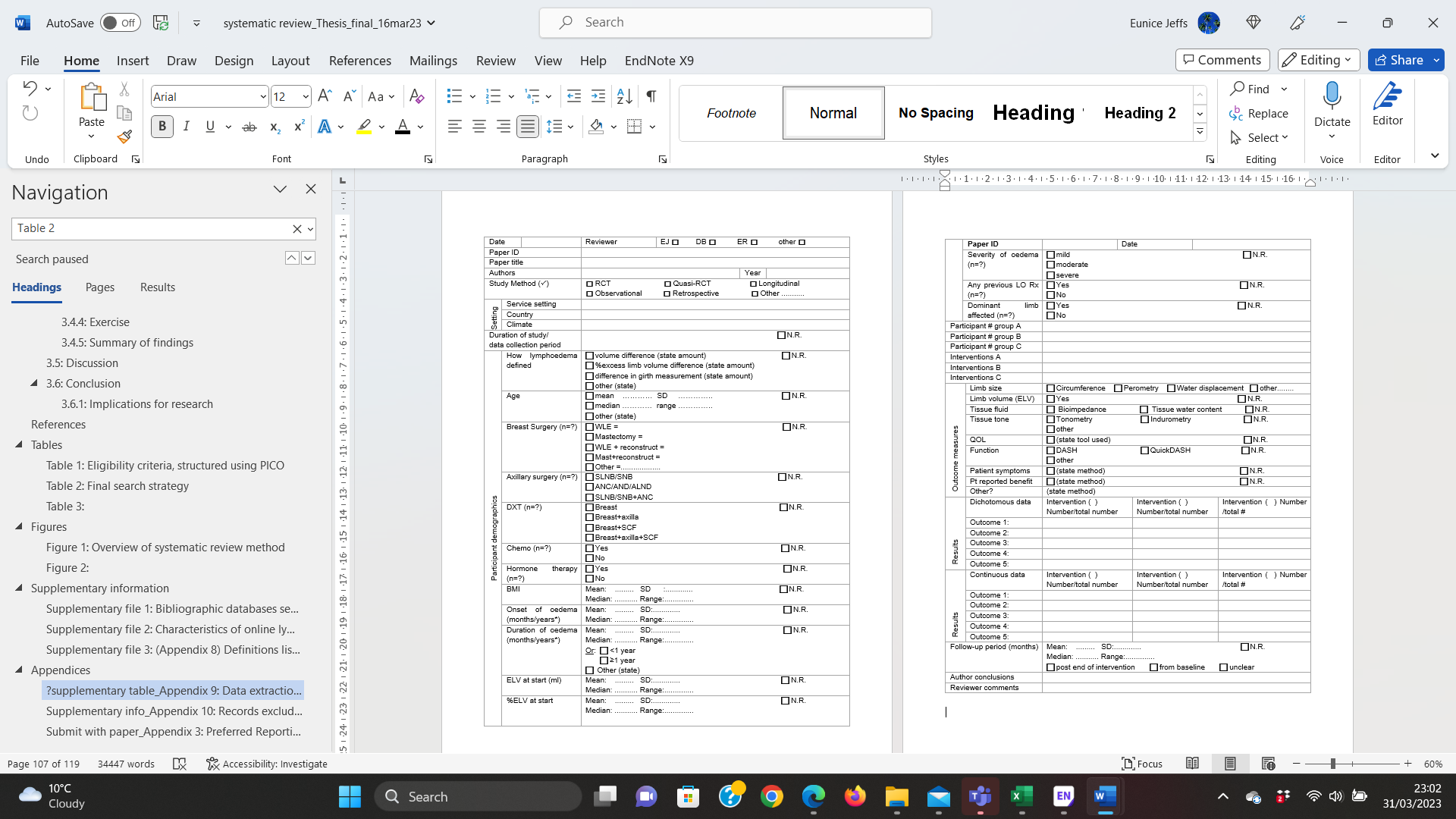


## Online Resource 7: Records excluded at full paper screening in 2018 and 2022

*Records excluded at full paper screening in original search (2016) are reported in Jeffs et al, 2018 [34].*

| Excluded: not the population of interest |
| --- |
| Aldrich, M. B., Gross, D., Morrow, J. R., Fife, C. E., & Rasmussen, J. C. (2017). Effect of pneumatic compression therapy on lymph movement in lymphedema-affected extremities, as assessed by near-infrared fluorescence lymphatic imaging. *Journal of Innovative Optical Health Sciences, 10*(2), 14. doi:10.1142/s1793545816500498  Lai, Y. T., Hsieh, C. C., Huang, L. S., Liu, W. S., Lin, S. H., Wang, L. L., Chen, S. F., Lin, C. C. (2017). The Effects of Upper Limb Exercise Through Yoga on Limb Swelling in Chinese Breast Cancer Survivors - A Pilot Study. *Rehabilitation Nursing Journal, 42*(1), 46-54. doi:10.1002/rnj.217  Li, K., Zhang, Z., Liu, N. F., Feng, S. Q., Tong, Y., Zhang, J. F., Constantinides, J., Lazzeri, D., Grassetti, L., Nicoli, F., Zhang, Y. X. (2017). Efficacy and safety of far infrared radiation in lymphedema treatment: clinical evaluation and laboratory analysis. *Lasers in Medical Science, 32*(3), 485-494. doi:10.1007/s10103-016-2135-0  Ozsoy-Unubol, T., Sanal-Toprak, C., Bahar-Ozdemir, Y., & Akyuz, G. (2019). Efficacy of kinesio taping in early stage breast cancer associated lymphedema: A randomized single blinded study. *Lymphology, 52*(4), 166-176. |

| Excluded: BCRL duration unknown |
| --- |
| Ali, K. M., El Gammal, E. R., & Eladl, H. M. (2021). Effect of Aqua Therapy Exercises on Postmastectomy Lymphedema: A Prospective Randomized Controlled Trial. *Ann Rehabil Med, 45*(2), 131-140. doi:10.5535/arm.20127  Argenbright, C. A., Taylor-Piliae, R. E., & Loescher, L. J. (2016). Bowenwork for symptom management of women breast cancer survivors with lymphedema: A pilot study. *Complementary Therapies in Clinical Practice, 25*, 142-149. doi:10.1016/j.ctcp.2016.09.004  Anand, B., Kalra, R., & Sharma, H. (2020). Effect of CDT and IPC in Secondary Upper Extremity Lymphedema in Women Post Breast Cancer Surgery on Anxiety, Depression and Quality of Life. *Indian Journal of Physiotherapy & Occupational Therapy, 14*(4), 211-216. doi:10.37506/ijpot.v14i4.11329  Basha, M. A., Aboelnour, N. H., Alsharidah, A. S., & Kamel, F. H. (2022). Effect of exercise mode on physical function and quality of life in breast cancer-related lymphedema: a randomized trial. *Supportive Care in Cancer, 30*(3), 2101-2110. doi:10.1007/s00520-021-06559-1  Blom, K. Y., Johansson, K. I., Nilsson-Wikmar, L. B., & Brogårdh, C. B. (2022). Early intervention with compression garments prevents progression in mild breast cancer-related arm lymphedema: a randomized controlled trial. *Acta Oncologica, 61*(7), 897-905. doi:10.1080/0284186X.2022.2081932  Deacon, R., de Noronha, M., Shanley, L., & Young, K. (2019). Does the speed of aquatic therapy exercise alter arm volume in women with breast cancer related lymphoedema? A cross-over randomized controlled trial. *Braz J Phys Ther, 23*(2), 140-147. doi:10.1016/j.bjpt.2018.11.004  Dhar, A., Srivastava, A., Pandey, R. M., Shrestha, P., Villet, S., & Rekha Gogia, A. (2022). Safety and Efficacy of a Mobiderm Compression Bandage During Intensive Phase of Decongestive Therapy in Patients with Breast Cancer-Related Lymphedema: A Randomized Controlled Trial. *Lymphat Res Biol*. doi:10.1089/lrb.2021.0104  Donahue, P. M., Crescenzi, R., Scott, A. O., Braxton, V., Desai, A., Smith, S. A., Jordi, J., Meszoely, I. M., Grau, A. M., Kauffmann, R. M., Sweeting, R. S., Spotanski, K., Ridner, S. H., Donahue, M. J. (2017). Bilateral Changes in Deep Tissue Environment After Manual Lymphatic Drainage in Patients with Breast Cancer Treatment-Related Lymphedema. *Lymphat Res Biol, 15*(1), 45-56. Doi:10.1089/lrb.2016.0020  Donmez, A. A., & Kapucu, S. (2017). The effectiveness of a clinical and home-based physical activity program and simple lymphatic drainage in the prevention of breast cancer-related lymphedema: A prospective randomized controlled study. *Eur J Oncol Nurs, 31*, 12-21. Doi:10.1016/j.ejon.2017.09.004  Jiang, L., Xie, N., & Ruan, X. (2018). Effects of pressure cuff assisted lymphatic drainage on upper limb lymphedema in patients after breast cancer surgery. *Chinese Journal of Rehabilitation Medicine, 33*(4), 409-412. Doi:10.3969/j.issn.1001-1242.2018.04.007  Kizil, R., Dilek, B., Sahin, E., Engin, O., Soylu, A. C., Akalin, E., & Alper, S. (2018). Is Continuous Passive Motion Effective in Patients with Lymphedema? A Randomized Controlled Trial. *Lymphat Res Biol, 16*(3), 263-269. Doi:10.1089/lrb.2017.0018  Lee, Q., Lee, Q., Stone, s., 李, 芹., & 石, 帆. (2021). 改良版综合消肿疗法治疗乳腺癌 相关淋巴水肿的效果观察. (Effects of pressure cuff-assisted lymphatic drainage on upper limb lymphedema in patients after breast cancer surgery) *Chinese Journal of Integrative Nursing, 7*(3), 107-109. doi:10.12209/j.issn2618-0219.202012181  Luz, R. P. C., Simao Haddad, C. A., Rizzi, S., Elias, S., Nazario, A. C. P., & Facina, G. (2018). Complex Therapy Physical alone or Associated with Strengthening Exercises in Patients with Lymphedema after Breast Cancer Treatment: a Controlled Clinical Trial. *Asian Pacific Journal of Cancer Prevention: Apjcp, 19*(5), 1405-1410. Doi:10.22034/apjcp.2018.19.5.1405  Meng, W. U., Bei, W., Yanyan, L., & Meijie, C. (2019). Application of manual lymph drainage operated by one person in patients with breast cancer-related lymphedema. *Nursing of Integrated Traditional Chinese & Western Medicine, 5*(9), 102-106. doi:10.11997/nitcwm.201909026  Mirandola, D., Monaci, M., Miccinesi, G., Muraca, M. G., Papi, F., Manetti, M., Sgambati, E., Marini, M. (2018). Effects of a specific adapted upper limb exercise on lymphedema in breast cancer survivors: A pilot study. *Gazzetta Medica Italiana Archivio per le Scienze Mediche, 177*(5), 204-211. Doi:http://dx.doi.org/10.23736/S0393-3660.17.03591-4  Mobarakeh, Z. S., Mokhtari-Hesari, P., Lotfi-Tokaldany, M., Montazeri, A., Heidari, M., & Zekri, F. (2019). Combined decongestive therapy and reduction of pain and heaviness in patients with breast cancer-related lymphedema. *Support Care Cancer, 27*(10), 3805-3811. doi:10.1007/s00520-019-04681-9  Muñoz-Alcaraz, M. N., Pérula-de-Torres, L., Serrano-Merino, J., Jiménez-Vílchez, A. J., Olmo-Carmona, M. V., Muñoz-García, M. T., Bartolomé-Moreno, C., Oliván-Blázquez, B., Magallón-Botaya, R. (2020). Efficacy and efficiency of a new therapeutic approach based on activity-oriented proprioceptive antiedema therapy (TAPA) for edema reduction and improved occupational performance in the rehabilitation of breast cancer-related arm lymphedema in women: a controlled, randomized clinical trial. *BMC Cancer, 20*(1), 1074. doi:10.1186/s12885-020-07558-x  Park, M. W., Lee, S. U., Kwon, S., & Seo, K. S. (2019). Comparison Between the Effectiveness of Complex Decongestive Therapy and Stellate Ganglion Block in Patients with Breast Cancer-Related Lymphedema: A Randomized Controlled Study. *Pain Physician, 22*(3), 255-263.  Pereira de Godoy, A. C., Guerreiro Godoy, M. F., Pereira de Godoy, L. M., Pereira de Godoy, H. J., & Pereira de Godoy, J. M. (2021). Intensive Treatment for Upper Limb Lymphedema. *Cureus, 13*(9), e18026. doi:10.7759/cureus.18026  Schmitz, K. H., Troxel, A. B., Dean, L. T., DeMichele, A., Brown, J. C., Sturgeon, K., Zhang, Z., Evangelisti, M., Spinelli, B., Kallan, M. J., Denlinger, C., Cheville, A., Winkels, R. M. Chodosh, L., Sarwer, D. B. (2019). Effect of Home-Based Exercise and Weight Loss Programs on Breast Cancer-Related Lymphedema Outcomes Among Overweight Breast Cancer Survivors: The WISER Survivor Randomized Clinical Trial. *JAMA Oncol, 5*(11), 1605-1613. doi:10.1001/jamaoncol.2019.2109  Torres-Lacomba, M., Navarro-Brazález, B., Prieto-Gómez, V., Ferrandez, J. C., Bouchet, J. Y., & Romay-Barrero, H. (2020). Effectiveness of four types of bandages and kinesio-tape for treating breast-cancer-related lymphoedema: a randomized, single-blind, clinical trial. *Clin Rehabil, 34*(9), 1230-1241. doi:10.1177/0269215520935943 |

| Excluded: BCRL duration >12 months |
| --- |
| Arinaga, Y., Piller, N., Sato, F., Ishida, T., Ohtake, T., Kikuchi, K., Sato-Tadano, A., Tada, H., Miyashita, M. (2019). The 10-Min Holistic Self-Care for Patients with Breast Cancer-Related Lymphedema: Pilot Randomized Controlled Study. *Tohoku J Exp Med, 247*(2), 139-147. doi:10.1620/tjem.247.139  Arinaga, Y., Sato, F., Piller, N., Kakamu, T., Kikuchi, K., Ohtake, T., Sakuyama, A., Yotsumoto, F., Hori, T., Sato, N. (2016). A 10 minute self-care program may reduce breast cancer-related lymphedema: A six-month prospective longitudinal comparative study. *Lymphology, 49*(2), 93-106.  Aykac Cebicci, M., & Dizdar, M. (2021). A comparison of the effectiveness of complex decongestive therapy and extracorporeal shock wave therapy in the treatment of lymphedema secondary to breast cancer. *Indian Journal of Surgery, 83*(3), 749-753. doi:10.1007/s12262-021-02769-3  Basoglu, C., Sindel, D., Corum, M., & Oral, A. (2021). Comparison of complete decongestive therapy and kinesiology taping for unilateral upper limb breast cancer-related lymphedema: A randomized controlled trial. *Lymphology, 54*(1), 41-51.  Borman, P., Yaman, A., Yasrebi, S., Pınar İnanlı, A., & Arıkan Dönmez, A. (2022). Combined Complete Decongestive Therapy Reduces Volume and Improves Quality of Life and Functional Status in Patients With Breast Cancer-Related Lymphedema. *Clinical Breast Cancer, 22*(3), e270-e277. doi:10.1016/j.clbc.2021.08.005  Buchan, J., Janda, M., Box, R., Schmitz, K., & Hayes, S. (2016). A Randomized Trial on the Effect of Exercise Mode on Breast Cancer-related Lymphedema. *Medicine & Science in Sports & Exercise*. doi:10.1249/mss.0000000000000988  Cacchio, A., Prencipe, R., Bertone, M., De Benedictis, L., Taglieri, L., D'Elia, E., Centoletti, C., Di Carlo, G. (2019). Effectiveness and safety of a product containing diosmin, coumarin, and arbutin (Linfadren®) in addition to complex decongestive therapy on management of breast cancer-related lymphedema. *Support Care Cancer, 27*(4), 1471-1480. doi:10.1007/s00520-018-4514-5  Cansız, G., Arıkan Dönmez, A., Kapucu, S., & Borman, P. (2022). The effect of a self-management lymphedema education program on lymphedema, lymphedema-related symptoms, patient compliance, daily living activities and patient activation in patients with breast cancer-related lymphedema: A quasi-experimental study. *European Journal of Oncology Nursing, 56*, N.PAG-N.PAG. doi:10.1016/j.ejon.2021.102081  Corum, M., Basoglu, C., Korkmaz, M. D., Yildirim, M. A., & Ones, K. (2021). Effectiveness of Combined Complex Decongestive Therapy and Resistance Exercises in the Treatment of Lymphedema Associated with Breast Cancer and the Effect of Pain on Treatment Response. *Lymphat Res Biol, 19*(4), 383-390. doi:10.1089/lrb.2020.0099  De Vrieze, T., Gebruers, N., Nevelsteen, I., Fieuws, S., Thomis, S., De Groef, A., Tjalma, W. A., Belgrado, J. P., Vandermeeren, L., Monten, C., Hanssens, M., Devoogdt, N. (2022). Manual lymphatic drainage with or without fluoroscopy guidance did not substantially improve the effect of decongestive lymphatic therapy in people with breast cancer-related lymphoedema (EFforT-BCRL trial): a multicentre randomised trial. *J Physiother, 68*(2), 110-122. doi:10.1016/j.jphys.2022.03.010  Duyur Cakıt, B., Pervane Vural, S., & Ayhan, F. F. (2019). Complex Decongestive Therapy in Breast Cancer-Related Lymphedema: Does Obesity Affect the Outcome Negatively? *Lymphat Res Biol, 17*(1), 45-50. doi:10.1089/lrb.2017.0086  Forner-Cordero, I., Muñoz-Langa, J., DeMiguel-Jimeno, J. M., & Rel-Monzó, P. (2021). Physical therapies in the decongestive treatment of lymphedema: A randomized, non-inferiority controlled study. *Clin Rehabil, 35*(12), 1743-1756. doi:10.1177/02692155211032651  Hemmati, M., Rojhani-Shirazi, Z., Zakeri, Z. S., Akrami, M., & Salehi Dehno, N. (2022). The effect of the combined use of complex decongestive therapy with electrotherapy modalities for the treatment of breast cancer-related lymphedema: a randomized clinical trial. *BMC Musculoskeletal Disorders, 23*(1), 1-8. doi:10.1186/s12891-022-05780-1  Keskin, D., Dalyan, M., Ünsal-Delialioğlu, S., & Düzlü-Öztürk, Ü. (2020). The results of the intensive phase of complete decongestive therapy and the determination of predictive factors for response to treatment in patients with breast cancer related-lymphedema. *Cancer Rep (Hoboken), 3*(2), e1225. doi:10.1002/cnr2.1225  Kilmartin, L., Denham, T., Fu, M. R., Yu, G., Kuo, T. T., Axelrod, D., & Guth, A. A. (2020). Complementary low-level laser therapy for breast cancer-related lymphedema: a pilot, double-blind, randomized, placebo-controlled study. *Lasers Med Sci, 35*(1), 95-105. doi:10.1007/s10103-019-02798-1  Koo, J. H., Song, S. H., Oh, H. S., & Oh, S. H. (2020). Comparison of the short-term effects of hyperbaric oxygen therapy and complex decongestive therapy on breast cancer-related lymphedema: A pilot study. *Medicine (Baltimore), 99*(11), e19564. doi:10.1097/md.0000000000019564  Kostanoglu, A., & Tarakcı, E. (2021). Physical therapy enhances functions and quality of life in older patients with breast cancer-related lymphedema: A prospective experimental study. *Niger J Clin Pract, 24*(3), 387-392. doi:10.4103/njcp.njcp_373_19  Lampinen, R., Lee, J. Q., Leano, J., Miaskowski, C., Mastick, J., Brinker, L., Topp, K., Smoot, B. (2021). Treatment of Breast Cancer-Related Lymphedema Using Negative Pressure Massage: A Pilot Randomized Controlled Trial. *Arch Phys Med Rehabil, 102*(8), 1465-1472.e1462. doi:10.1016/j.apmr.2021.03.022  Lee, K. W., Kim, S. B., Lee, J. H., & Kim, Y. S. (2020). Effects of Extracorporeal Shockwave Therapy on Improvements in Lymphedema, Quality of Life, and Fibrous Tissue in Breast Cancer-Related Lymphedema. *Ann Rehabil Med, 44*(5), 386-392. doi:10.5535/arm.19213  Liang, X., You, M., Wen, C., Hou, F., Kang, J., Lv, Z., & Tian, J. (2022). Self-administration of complex decongestive therapy facilitated by the mobile application WeChat improves lymphedema and quality of life in breast cancer survivors: an observational study. *Ann Transl Med, 10*(3), 146. doi:10.21037/atm-21-6662  Ligabue, M. B., Campanini, I., Veroni, P., Cepelli, A., Lusuardi, M., & Merlo, A. (2019). Efficacy of self-administered complex decongestive therapy on breast cancer-related lymphedema: a single-blind randomized controlled trial. *Breast Cancer Res Treat, 175*(1), 191-201. doi:10.1007/s10549-019-05136-9  Loudon, A., Barnett, T., Piller, N., Immink, M., Visentin, D., & Williams, A. (2016). The effects of yoga on shoulder and spinal actions for women with breast cancer-related lymphoedema of the arm: a randomised controlled pilot study [Randomized Controlled Trial]. *BMC Complementary and Alternative Medicine, 16*, 343. doi:10.1186/s12906-016-1330-7. (Accession No. CN-01263378)  McNeely, M. L., Dolgoy, N. D., Rafn, B. S., Ghosh, S., Ospina, P. A., Al Onazi, M. M., Radke, L., Shular, M., Kuusk, U., Webster, M., Campbell, K. L., Mackey, J. R. Mackey, J. R. (2022). Nighttime compression supports improved self-management of breast cancer-related lymphedema: A multicenter randomized controlled trial. *Cancer, 128*(3), 587-596. doi:10.1002/cncr.33943  Mestre, S., Calais, C., Gaillard, G., Nou, M., Pasqualini, M., Ben Amor, C., & Quere, I. (2017). Interest of an auto-adjustable nighttime compression sleeve (MOBIDERM(R) Autofit) in maintenance phase of upper limb lymphedema: the MARILYN pilot RCT. *Support Care Cancer, 25*(8), 2455-2462. Doi:10.1007/s00520-017-3652-5  Mestre, S., Gaillard, G., Benhamou, M., Soulier-Sotto, V., Nou, M., Pasqualini, M., Amor, C. B. Quere, I. (2017). An Auto-Adjustable Night Garment to Control Early Rebound Effect of Edema Volume After Intensive Phase of Decongestive Lymphedema Therapy. *Lymphat Res Biol, 15*(4), 364-370. Doi:10.1089/lrb.2017.0026  Omar, M. T. A., Gwada, R. F. M., Omar, G. S. M., El-Sabagh, R. M., & Mersal, A. A. E. (2020). Low-Intensity Resistance Training and Compression Garment in the Management of Breast Cancer-Related Lymphedema: Single-Blinded Randomized Controlled Trial. *J Cancer Educ, 35*(6), 1101-1110. doi:10.1007/s13187-019-01564-9  Osorio, F., Ferro, L., Garrido, L., Henriques, A., Cruz, J., Fangueiro, R., Fougo, J. L. Azevedo, A. (2017). Satisfaction with a therapeutic sleeve for arm lymphedema secondary to breast cancer treatment: Controlled crossover trial. *Porto Biomedical Journal, 2*(1), 13-17. doi:http://dx.doi.org/10.1016/j.pbj.2016.11.005  Ozcan, D. S., Dalyan, M., Unsal Delialioglu, S., Duzlu, U., Polat, C. S., & Koseoglu, B. F. (2018). Complex Decongestive Therapy Enhances Upper Limb Functions in Patients with Breast Cancer-Related Lymphedema. *Lymphat Res Biol*. Doi:10.1089/lrb.2017.0061  Pajero Otero, V., García Delgado, E., Martín Cortijo, C., Rodríguez Ramos, M. L., De Carlos Iriarte, E., Gil García, A., Romay-Barrero, H., Avendaño-Coy, J. (2022). Intensive complex physical therapy combined with intermittent pneumatic compression versus Kinesio taping for treating breast cancer-related lymphedema of the upper limb: A randomised cross-over clinical trial. *Eur J Cancer Care (Engl)*. doi:10.1111/ecc.13625  Pajero Otero, V., García Delgado, E., Martín Cortijo, C., Romay Barrero, H. M., de Carlos Iriarte, E., & Avendaño-Coy, J. (2019). Kinesio taping versus compression garments for treating breast cancer-related lymphedema: a randomized, cross-over, controlled trial. *Clin Rehabil, 33*(12), 1887-1897. doi:10.1177/0269215519874107  Park, J. H. (2017). The effects of complex exercise on shoulder range of motion and pain for women with breast cancer-related lymphedema: a single-blind, randomized controlled trial. *Breast Cancer, 24*(4), 608-614. doi:10.1007/s12282-016-0747-7  Pujol-Blaya, V., Salinas-Huertas, S., Catasús, M. L., Pascual, T., & Belmonte, R. (2019). Effectiveness of a precast adjustable compression system compared to multilayered compression bandages in the treatment of breast cancer-related lymphoedema: a randomized, single-blind clinical trial. *Clin Rehabil, 33*(4), 631-641. doi:10.1177/0269215518821785  Rockson, S. G., Whitworth, P. W., Cooper, A., Kania, S., Karnofel, H., Nguyen, M., Shadduck, K., Gingerich, P., Armer, J. (2022). Safety and Effectiveness of a Novel Non-Pneumatic Active Compression Device for Treating Breast Cancer-Related Lymphedema, a Multi-center Randomized, Crossover Trial (NILE). *J Vasc Surg Venous Lymphat Disord*. doi:10.1016/j.jvsv.2022.06.016  Sanal-Toprak, C., Ozsoy-Unubol, T., Bahar-Ozdemir, Y., & Akyuz, G. (2019). The efficacy of intermittent pneumatic compression as a substitute for manual lymphatic drainage in complete decongestive therapy in the treatment of breast cancer related lymphedema. *Lymphology, 52*(2), 82-91.  Sen, E. I., Arman, S., Zure, M., Yavuz, H., Sindel, D., & Oral, A. (2021). Manual Lymphatic Drainage May Not Have an Additional Effect on the Intensive Phase of Breast Cancer-Related Lymphedema: A Randomized Controlled Trial. *Lymphat Res Biol, 19*(2), 141-150. doi:10.1089/lrb.2020.0049  Tambour, M., Holt, M., Speyer, A., Christensen, R., & Gram, B. (2018). Manual lymphatic drainage adds no further volume reduction to Complete Decongestive Therapy on breast cancer-related lymphoedema: a multicentre, randomised, single-blind trial. *Br J Cancer, 119*(10), 1215-1222. doi:10.1038/s41416-018-0306-4  Tastaban, E., Soyder, A., Aydin, E., Sendur, O. F., Turan, Y., Ture, M., & Bilgen, M. (2020). Role of intermittent pneumatic compression in the treatment of breast cancer-related lymphoedema: a randomized controlled trial. *Clin Rehabil, 34*(2), 220-228. doi:10.1177/0269215519888792  Tatar, K. K., & Turhan, B. (2022). The effects of complex decongestive therapy on pain and functionality in individuals with breast cancer who developed adhesive capsulitis due to lymphedema: an evaluation by an isokinetic computerized system. *Korean J Pain, 35*(3), 280-290. doi:10.3344/kjp.2022.35.3.280  Whatley, J., Street, R., & Kay, S. (2018). Experiences of breast cancer related lymphoedema and the use of reflexology for managing swelling: A qualitative study. *Complementary therapies in clinical practice, 32*, 123-129. doi:10.1016/j.ctcp.2018.06.006  Yaman, A., Borman, P., İnanlı, A., Kul, F., & Karahan, S. (2021). The efficacy of different bandaging methods in patients with breast cancer-related lymphedema: A prospective, randomized study. *Turk J Phys Med Rehabil, 67*(2), 155-166. doi:10.5606/tftrd.2021.6287  Yesil, H., Eyigor, S., Caramat, I., & Isik, R. (2017). Effects of complex decongestive therapy on quality of life, depression, neuropathic pain, and fatigue in women with breast cancer-related lymphedema. *Turkiye Fiziksel Tip ve Rehabilitasyon Dergisi, 63*(4), 329-334. doi:http://dx.doi.org/10.5606/tftrd.2017.779  Yeşil, H., Eyigör, S., İnbat, M., & Bulut, F. (2021). The effects of complex decongestive therapy on kinesthetic sense of hands, upper extremity function, and quality of life in patients with breast cancer-related lymphedema. *Turk J Phys Med Rehabil, 67*(2), 211-217. doi:10.5606/tftrd.2021.5191  Zhang, X., Brown, J. C., Paskett, E. D., Zemel, B. S., Cheville, A. L., & Schmitz, K. H. (2017). Changes in arm tissue composition with slowly progressive weight-lifting among women with breast cancer-related lymphedema. *Breast Cancer Research & Treatment, 164*(1), 79-88. doi:10.1007/s10549-017-4221-9 |

| Excluded: not the outcome of interest |
| --- |
| Bozkurt, M., Palmer, L. J., & Guo, Y. (2017). Effectiveness of Decongestive Lymphatic Therapy in Patients with Lymphedema Resulting from Breast Cancer Treatment Regardless of Previous Lymphedema Treatment. *Breast J, 23*(2), 154-158. doi:10.1111/tbj.12710  Ergin, G., Şahinoğlu, E., Karadibak, D., & Yavuzşen, T. (2018). Effect of Bandage Compliance on Upper Extremity Volume in Patients with Breast Cancer-Related Lymphedema. *Lymphat Res Biol, 16*(6), 553-558. doi:10.1089/lrb.2017.0060  Ergin, G., Şahinoğlu, E., Karadibak, D., & Yavuzşen, T. (2019). Effectiveness of Kinesio Taping on Anastomotic Regions in Patients with Breast Cancer-Related Lymphedema: A Randomized Controlled Pilot Study. *Lymphat Res Biol, 17*(6), 655-660. doi:10.1089/lrb.2019.0003  Karafa, M., Karafova, A., & Szuba, A. (2018). The effect of different compression pressure in therapy of secondary upper extremity lymphedema in women after breast cancer surgery. *Lymphology, 51*(1), 28-37.  Kozanoglu, E., Gokcen, N., Basaran, S., & Paydas, S. (2022). Long-Term Effectiveness of Combined Intermittent Pneumatic Compression Plus Low-Level Laser Therapy in Patients with Postmastectomy Lymphedema: A Randomized Controlled Trial. *Lymphat Res Biol, 20*(2), 175-184. doi:10.1089/lrb.2020.0132  Pasyar, N., Barshan Tashnizi, N., Mansouri, P., & Tahmasebi, S. (2019). Effect of yoga exercise on the quality of life and upper extremity volume among women with breast cancer related lymphedema: A pilot study. *Eur J Oncol Nurs, 42*, 103-109. doi:10.1016/j.ejon.2019.08.008  Rodríguez Manso, M., Canosa Hermida, E., Martín Mourelle, R., Otero Villaverde, S., Mora Boga, R., & Balboa Barreiro, V. (2019). Terapia descongestiva compleja en grados iniciales de linfedema secundario a cáncer de mama. *Fisioterapia, 41*(1), 21-27. doi:10.1016/j.ft.2019.01.001  Storz, M. A., Gronwald, B., Gottschling, S., Schope, J., Mavrova, R., & Baum, S. (2017). Photobiomodulation therapy in breast cancer-related lymphedema: a randomized placebo-controlled trial. *Photodermatol Photoimmunol Photomed, 33*(1), 32-40. Doi:10.1111/phpp.12284  Chmielewska, D., Stania, M., Blaszczak, E., & Kwasna, K. (2016). Intermittent pneumatic compression in patients with postmastectomy lymphedema [Journal: Article]. *Family medicine and primary care review, 18*(4), 419-424. doi:10.5114/fmpcr.2016.63694. (Accession No. CN-01473727)  Darowska-Olszewska, J., & Śliwiński, Z. (2016). Evaluation of impact of the application of kinesio taping and standard complete decongestive therapy on the lymphedema in women after mastectomy. *Fizjoterapia Polska, 16*(3), 18-32. Retrieved from http://fizjoterapiapolska.pl/wp-content/uploads/2016/12/ENGLISH-FP32016-18-32.pdf  Qiang, W.-m., Wang, Y., Tang, L., & Shen, Y. O. L. (2016). *The effects of microwave therapy combined with physical therapy for breast cancer related lymphedema on involved upper arms Source: Zhonghua hu li za zhi = Chinese journal of nursing. 51, no. 5, (2016): 534-538* |

| Excluded: not the intervention of interest |
| --- |
| Bell, L. R. (2017). Effectiveness of self-lymphatic drainage? *Rehabilitation Oncology, 35*(2), 102-103. doi:http://dx.doi.org/10.1097/01.REO.0000000000000057  Do, J., Jeon, J., & Kim, W. (2017). The effects of bandaging with an additional pad and taping on secondary arm lymphedema in a patient after mastectomy. *J Phys Ther Sci, 29*(7), 1272-1275. doi:10.1589/jpts.29.1272  Godoy, d. F. G. G., M., Pereira de Godoy, A. C., & Pereira de Godoy, J. M. (2017). Effect of exercise while utilizing a device with an arm compression sleeve to reduce lymphedema. *Clin Exp Obstet Gynecol, 44*(1), 17-19.  Sapula, R., Braniewska, J., Weremczuk, R., Wolanin, M., & Sapula, J. (2017). The evaluation of selected physiotherapeutic methods in the treatment of post-mastectomy lymphoedema. *Postepy Rehabilitacji, 31*(2), 5-15. doi:http://dx.doi.org/10.1515/rehab-2015-0063 |
